# Development and implementation of a centralized surveillance infection prevention program in a multi-facility health system: A quality improvement project

**DOI:** 10.1017/ash.2023.126

**Published:** 2023-03-22

**Authors:** Graham M. Snyder, Suzanne Wagester, Patricia L. Harris, Abby L. Valek, Jacob C. Hodges, Andrew L. Bilderback, Fazrina Kader, Colleen A. Tanner, Amy P. Metzger, Susan E. DiNucci, Bonnie V. Colaianne, Ashley Chung, Rachel L. Zapf, Paula L. Kip, Tamra E. Minnier

**Affiliations:** 1 Department of Infection Prevention and Control, UPMC Presbyterian/Shadyside, Pittsburgh, Pennsylvania; 2 Division of Infectious Diseases, University of Pittsburgh School of Medicine, Pittsburgh, Pennsylvania; 3 Wolff Center, UPMC, Pittsburgh, Pennsylvania; 4 Quality and Risk Management, UPMC Passavant, McCandless, Pennsylvania

## Abstract

**Objective:**

To develop, implement, and evaluate the effectiveness of a unique centralized surveillance infection prevention (CSIP) program.

**Design:**

Observational quality improvement project.

**Setting:**

An integrated academic healthcare system.

**Intervention:**

The CSIP program comprises senior infection preventionists who are responsible for healthcare-associated infection (HAI) surveillance and reporting, allowing local infection preventionists (LIPs) a greater portion of their time to non-surveillance patient safety activities. Four CSIP team members accrued HAI responsibilities at 8 facilities.

**Methods:**

We evaluated the effectiveness of the CSIP program using 4 measures: recovery of LIP time, efficiency of surveillance activities by LIPs and CSIP staff, surveys characterizing LIP perception of their effectiveness in HAI reduction, and nursing leaders’ perception of LIP effectiveness.

**Results:**

The amount of time spent by LIP teams on HAI surveillance was highly variable, while CSIP time commitment and efficiency was steady. Post-CSIP implementation, 76.9% of LIPs agreed that they spend adequate time on inpatient units, compared to 15.4% pre-CSIP; LIPs also reported more time to allot to non-surveillance activities. Nursing leaders reported greater satisfaction with LIP involvement with HAI reduction practices.

**Conclusion:**

CSIP programs are a little-reported strategy to ease burden on LIPs with reallocation of HAI surveillance. The analyses presented here will aid health systems in anticipating the benefit of CSIP programs.

Healthcare-associated infections (HAIs) are a serious healthcare concern and negatively impact the health and safety of patients and staff. Approximately 4% of hospitalized patients will develop HAI and, of these, ∼11% will die during hospitalization.^1,2^ Common HAIs include central line–associated bloodstream infections (CLABSIs), catheter-associated urinary tract infections (CAUTIs), gastrointestinal infections caused by *Clostridioides difficile*, and surgical site infections (SSIs).^3–7^

HAI prevention in healthcare facilities is typically led by 1 or more local infection preventionists (LIPs) who work onsite at a single facility, in partnership with nursing colleagues as well as other healthcare workers. Although not systematically reported in the literature, LIP duties are often heavily focused on HAI surveillance and reporting, which reduces their availability for harm-reduction activities such as unit-based rounding, collaborative projects, professional development, and other activities. This lack of balance leads to decreased job satisfaction and increased turnover rates, as well as slower progress in HAI prevention initiatives.^8^ Published reports of alternative models of HAI surveillance including centralized surveillance infection prevention (CSIP) programs are limited, and no studies have characterized the relationship between CSIP models of HAI surveillance and improved infection-related patient outcomes.^9^

We developed and implemented a CSIP program that includes a team of system-level infection preventionists who undertake responsibility of HAI surveillance and reporting and therefore allow LIPs to refocus their time on harm reduction efforts. The aims of this quality improvement work were to demonstrate recovery of LIP time, quantify the efficiency of surveillance activities by LIPs and CSIP infection preventionists. We characterized LIP perceptions of their effectiveness in HAI reduction using a mixed-method survey, and we evaluated nursing leaders’ perceptions of LIP effectiveness in HAI reduction using pre- and post-intervention surveys.

## Methods

### Design

This quality improvement intervention was granted approval as a quality improvement project by an internal quality improvement review committee (project no. 1905). All methods and results are reported in accordance with Strengthening the Reporting of Observational Studies in Epidemiology (STROBE) statement and Standards for Quality Improvement Reporting Excellence (SQUIRE) guidelines (Supplementary Checklist 1).^10,11^ Web-based surveys were conducted in accordance with the guidelines set forth in the Checklist for Reporting Results of Internet E-Surveys (CHERRIES) (Supplementary Checklist 2).^12^

### Setting

Observations and data collection for this quality improvement initiative were conducted beginning January 2019 and concluding December 2021 at a 40-hospital integrated academic healthcare system. The CSIP program of HAI surveillance was implemented sequentially and at operationally convenient intervals among 12 acute care facilities over the 3-year period reported here.

In this analysis, all facilities implementing CSIP programs during the 4-year analysis period were assigned identifiers beginning with C (denoting a CSIP facility) and ending with a number assigned in order of the date of CSIP implementation. In total, 6 facilities implemented CSIP in 2019 (C1, C2, C3, C4, C5, C6), 2 facilities implemented CSIP in 2020 (C7, C8), and 4 facilities implemented CSIP in 2021 (C9, C10, C11, C12). Adoption of the CSIP program was nonrandom; it was developed and first initiated in large urban facilities with complex patient care populations (C1 and C2) and was subsequently adopted based on operational needs and staffing capacity. The 12 facilities in this analysis were variable in both location (serving urban, rural, and suburban populations) and size. One facility had <100 beds, 6 facilities had 100–300 beds, 4 facilities had 301–500 beds, and 1 facility had >500 beds. Also, 6 facilities had 1 LIP, 4 facilities had 2–4 LIPs, 1 facility had 7 LIPs, and 1 facility had 11 LIP personnel.

### Intervention

**Fig. 1.**
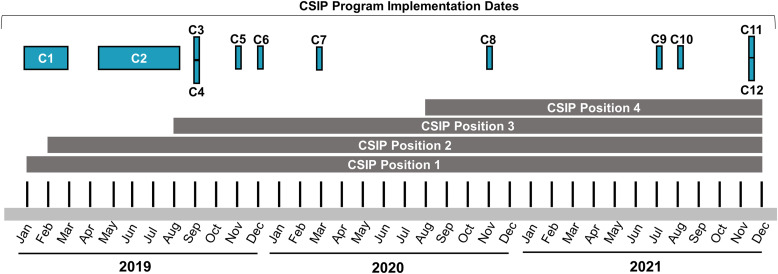
Centralized surveillance infection prevention (CSIP) project and analysis timeline.

The CSIP program comprises senior infection preventionists performing HAI surveillance and data analyses for facilities adopting centralized surveillance (Fig. 1). CSIP program infection preventionists are hired from LIP and non-LIP positions within the organization, as well as individuals from other organizations.

### Outcomes and data sources

We evaluated the implementation and effectiveness of the CSIP program using 4 measures: recovery of LIP time, efficiency of surveillance activities by LIPs and CSIP infection preventionists, and pre- and post-intervention mixed methods surveys characterizing both LIP perception of their effectiveness in HAI reduction and nursing leaders’ perception of LIP effectiveness in HAI reduction.

Prior to this quality improvement intervention, LIP time spent performing HAI surveillance was not quantified. Beginning measurement at least 1 month prior to transition from LIP to CSIP program surveillance, LIPs at each facility recorded the hours they spent daily performing HAI surveillance until surveillance was no longer performed by LIPs at that facility. CSIP team members also recorded daily hours spent performing HAI surveillance for the duration of this assessment. Hours spent performing surveillance were recorded by individual infection preventionists, reported to their direct supervisor, and aggregated anonymously.

To quantify surveillance efficiency of LIPs and CSIPs, 2 measures were taken: (1) the number of microbiology results (positive cultures) reviewed by IPs per hour of work, and (2) the number of HAI that were reported per hour of work. CSIP efficiency was measured weekly 6 weeks before program implementation to 100 weeks after implementation. Facility LIP efficiency was measured during the time LIPs measured hours performing surveillance. Surveillance measures were collected from the facilities’ surveillance software (TheraDoc Clinical Surveillance, TheraDoc, Charlotte NC).

To estimate the perceived use and effectiveness of reallocated LIP time from surveillance to other infection risk reduction efforts, email-based surveys were sent to LIPs and nursing unit directors at facilities before and after the implementation of the CSIP program. LIPs were asked how many minutes they spent in person on in-patient and ancillary units per month, how rewarding infection prevention tasks were, and about their perceived ability to attain quality and professional goals. The pre- and post-CSIP surveys were the same, except 1 additional question after CSIP implementation asking which tasks were allocated more time and effort after CSIP adoption. If an LIP did not complete a pre-CSIP program survey, they were not invited to complete a post-CSIP program implementation survey. Due to time and staffing constraints during the COVID-19 pandemic, only staff at facilities C1–C6 were contacted to complete these surveys.

Unit directors were asked before and after the CSIP intervention whether they were satisfied with the infection prevention duties of the LIP partnering with that unit, including time spent on the unit, knowledge, and impact on patient care. Those who responded to the first set of surveys were contacted ∼8 months after completion of the pre-CSIP survey to complete the same survey after CSIP program implementation (Supplementary Table S1). Surveys of LIPs and unit directors were conducted using a secure web-based tool (REDCap) and were voluntary and confidential.^13^ HAI definitions and reporting were conducted following NHSN standards.^14^

### Statistical analysis methods

LIP and CSIP weekly surveillance hours and HAI surveillance efficiency are displayed as descriptive line charts, aggregated into weekly measurements. LIP and unit director survey responses are presented as descriptive analyses.

## Results

Over the course of this quality improvement project, 4 infection preventionist positions were filled to serve as CSIP team members and 8 facilities transitioned HAI surveillance from LIPs to the CSIP program; 4 facilities transitioned during the quality improvement project but after evaluation of outcomes described here were concluded (Fig. 1).

### Recovery of LIP time and LIP and CSIP surveillance efficiency

**Fig. 2.**
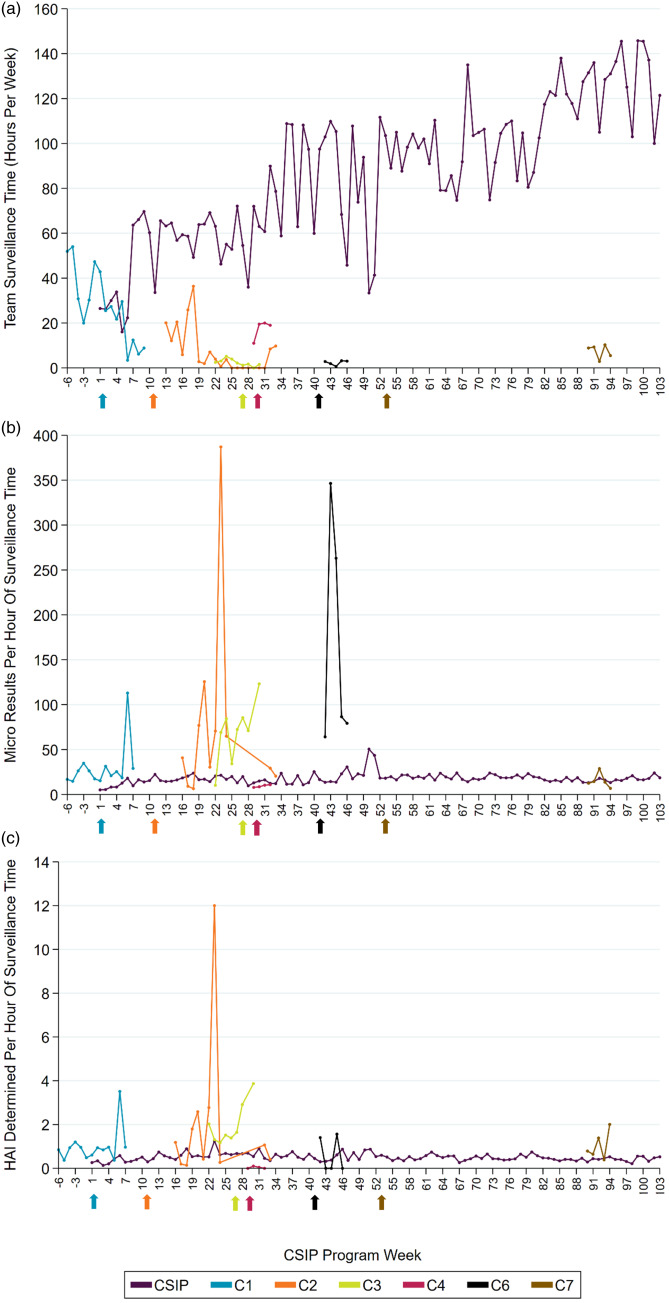
Measures of HAI surveillance time and surveillance efficiency for LIP facilities and CSIP program, by week of CSIP program implementation. The arrows indicate when each facility implemented CSIP. Arrows are color-coded specific to each facility as described in the key. (a) Measure of team HAI surveillance hours, by CSIP program week. (b) Microbiology results viewed per hour of surveillance time, by CSIP program week. (c) HAI determined per hour of surveillance time, by CSIP program week. Note. CSIP, centralized surveillance infection prevention; LIP, local infection preventionist.

The average hours per LIP per week performing HAI surveillance at all facilities was 6.5 (median hours, 0; IQR, 0–7.1). In the 103 weeks after surveillance performed by LIPs at 8 facilities was taken over by the CSIP program, 4 CSIPs spent an average of 87.1 hours per week (median hours, 91; IQR, 63.2–108.5) on HAI surveillance. LIPs averaged 167 microbiology results reviewed weekly (median, 0 results; IQR, 0–260) and 4.7 HAI reported weekly (median 0, HAI; IQR, 0–6.5). After the first facility completed the HAI surveillance transition to CSIP infection preventionists, CSIP infection preventionists’ averaged 1,494 microbiology results reviewed weekly (median, 1,695 results; IQR, 1,066.5–1,906.5) and 41.6 HAI reported weekly (median, 41 HAIs; IQR, 34.5–50) (Fig. 2). All LIPs conduct surveillance and review microbiology results. However, workload varies weekly, and surveillance and microbiology result review are not carried out every week. CSIP staff members are not assigned to specific hospitals; rather, CSIPs are assigned work based on patient acuity and volume.

### Perceived effectiveness of CSIP program: LIP and unit director surveys

Pre-CSIP implementation surveys were sent to 16 LIPs and 83 unit directors across 6 acute care facilities; 15 LIPs responded (93.76%) and 57 (68.67%) unit directors responded. Post-CSIP implementation surveys were sent to 14 LIPs and 57 unit directors; 12 LIPs responded (85.71%) and 32 unit directors responded (56.14%) (Table 1).

**Table 1a.** Local Infection Preventionist (LIP) and Unit Director Survey Responses Before and After CSIP Implementation LIP Survey Responses^a^

Survey Question	Before CSIP Implementation (N = 13), No. (%)	After CSIP Implementation (N = 13), No. (%)
**Time allocation^b^**		
I spend an adequate amount of time per month on my assigned inpatient units	2 (15.38)	10 (76.92)
I spend an adequate amount of time per month on my assigned ancillary units.	2 (15.38)	6 (46.15)
I am a knowledgeable resource for providers in my assigned areas.	10 (76.92)	11 (84.62)
I am ________ with my involvement in infection control practices in my assigned areas.	4 (30.77)	7 (53.85)
Infection prevention task satisfaction^c^		
**Rate the IP responsibilities according to how rewarding you find the following tasks, median, IQR**		
HAI surveillance competency work	63 (53–70)	50 (40–71)
Unit rounding	70 (65–89)	75 (60–82)
Root-cause analyses and case reviews	75 (60–80)	57.5 (46.25–76.75)
Attending meetings	75 (61–85)	60 (50–75)
Phone calls from external customers (eg, patients, families, DOH, vendors)	51 (38–65)	50 (40–70)
Phone calls from internal customers (eg, unit directors, nurses, EVS, physicians)	75 (55–80)	65 (60–75)
Individual projects	88 (77–93)	80 (50–90)
Collaborative projects with other departments	85 (80–91)	78 (73–85)
Urgent/emergent situations	83 (76–85)	71 (38–80)
Education (eg, RN/PCT orientation, unit-based)	85 (70–93)	70 (51–90)
Regulatory tasks (eg, Joint Commission tracers/audits)	50 (50–50)	36 (30–70)
**Quality and professional goal^b^**	No. (%)	No. (%)
As an IP, I have a positive impact on the health system as an organization.	11 (84.62)	8 (61.54)
My job makes good use of my skills and abilities.	12 (92.31)	8 (61.54)
I have clearly defined professional goals.	9 (69.23)	6 (46.15)
I have enough time to achieve my professional goals.	7 (53.85)	6 (46.15)
I have clearly defined quality goals.	9 (69.23)	8 (64.54)
I have enough time to achieve my quality goals.	6 (46.15)	6 (46.15)
My quality goals are oriented toward improving patient care.	13 (100)	10 (76.92)

Note: CSIP, centralized surveillance infection prevention; IP, infection preventionist; IQR, interquartile range; HAI, healthcare-associated infection; DOH, Department of Health; EVS, environmental services; RN, registered nurse; PCT, patient care technician.

a Data are only from individuals who completed both the pre- and post-CSIP surveys. Data from all individuals who completed either survey are available in Supplementary Table S2.

b Percentage reporting “agree” or “strongly agree”, or “satisfied” or “very satisfied.”

c Median (interquartile range) response, with 0 representing “not rewarding,” 50 representing “somewhat rewarding,” and 100 representing “very rewarding.”

**Table 1b.** Unit Director Survey Responses^a^

Survey Question^b^	Pre-CSIP Implementation (N = 34), No. (%)	Post-CSIP Implementation (N = 34), No. (%)
The IP assigned to my unit spends an adequate amount of time per month on the inpatient unit.	17 (50)	23 (67.65)
The IP assigned to my unit is a knowledgeable resource for the providers on the inpatient unit.	33 (97)	30 (88.24)
The IP assigned to my unit has played an important role in improving patient care on the inpatient unit.	24 (70.59)	26 (76.47)
Participating in environmental rounding with my IP is value added.	28 (82.35)	30 (88.24)
I am _________ with my IP’s involvement in infection control practices on my inpatient unit.	29 (85.29)	31 (91.18)

Note: CSIP, centralized surveillance infection prevention; IP, infection preventionist.

a Data presented in this table are only from individuals who completed both the pre- and post-CSIP surveys. Data from all individuals who completed either survey are available in Supplementary Table S2.

b Percentage reporting “agree” or “strongly agree” or “satisfied” or “very satisfied.”

The pre- and post-CSIP survey responses from LIPs related to their perceived resourcefulness, rewarding nature of work, and ability to accomplish quality and professional goals are presented in Table 1a. After CSIP implementation, LIPs reported most frequently spending more time on increasing their presence on assigned inpatient units (10 of 13, 76.9%), participating in additional collaborative projects (9 of 13, 69.2%), and attending meetings (9 of 13, 69.2%).

The pre- and post-CSIP survey responses from unit directors related to their perceived resourcefulness of LIPs are presented in Table 1b. Prior to CSIP implementation, unit directors reported that LIPs could be more effective at improving patient care by increasing time spent on the inpatient unit (17 of 34, 50.0%). Among the 34 unit directors, 29 (85.29%) were satisfied with LIP involvement in IP work. Following CSIP implementation, 23 unit directors (67.65%) were satisfied that LIPs spend an adequate amount of time on inpatient units, and 31 (91.18%) were satisfied with LIP involvement in IP work. Full results of the LIP and unit director surveys are reported in Supplementary Table S2 (all survey respondents), Supplementary Figure S1 (reallocation of LIP time), Supplementary Table S3 (LIP surveys), and Supplementary Table S4 (unit director surveys). A survey of LIPs regarding the perceived impact of COVID-19 on HAI reduction work, performed as a post-hoc analysis, is presented in Supplementary Figures S2–S4.

## Discussion

In this quality improvement intervention characterizing the implementation of a CSIP program at 12 acute care facilities, we identified several major themes: LIP time and efficiency spent performing surveillance was more variable than CSIP time and efficiency. Both LIPs and unit directors perceived significant improvement in LIP ability to reduce infection-related patient harm after adoption of CSIP. These observations are a substantial contribution to the published experience of centralized surveillance. They will be important to multiple-facility health systems considering a centralized model for HAI surveillance, and they describe a model that may improve patient safety in acute care settings.^9,15^

When reporting on LIP and CSIP efficiency and activities, there are some limitations. There are no robust measures of time and efficiency; efficiency was lower than we anticipated for CSIP infection preventionists and more variable than we anticipated for LIPs. We believe that focusing work activity on the surveillance task provides our organization with a more reliable and accurate HAI reporting system. Anecdotally, LIPs reported that HAI surveillance was often sporadic (eg, concentrated and rushed to meet a reporting deadline), which may account for spikes in efficiency rates. LIPs still review reported HAIs and are in close communication with CSIP infection preventionists about time-sensitive findings. Future studies should investigate whether LIPs vs CSIPs have more “accurate” HAI reporting. Additionally, CSIP programs may have a better ability to detect transmission involving multiple facilities, although we cannot yet evaluate that.

With implementation of the CSIP program, LIPs saw greater opportunities to effect change with less of their time allocated to HAI surveillance. However, LIPs reported unchanged or lower satisfaction with professional goals and sense of rewarding work after CSIP implementation. This finding contrasts with the responses of unit directors, which reported similar or improved opinions of LIP work after CSIP implementation. We attribute this reported dissatisfaction to the dramatic change in their roles after CSIP implementation. LIPs content with technical HAI surveillance work no longer had this work that they had perceived as gratifying, and many LIPs did not feel fully prepared to play a more prominent role in quality improvement work or to be responsible for seeking innovative solutions. The ongoing COVID-19 pandemic also prevented the collection of a larger survey cohort for LIP and UD surveys, or repeated surveys. However, overall, organizational assessment of the CSIP program in our health system has concluded that it is a positive change to HAI surveillance and reporting.

With the evolving role of infection preventionists, levels of work-related stress may be rising. CSIP programs may allow them to focus on the interdisciplinary and outcome-oriented activities, which may in turn improve effectiveness and job satisfaction.^16^ Because of the perceived benefit of CSIP programs and the data observed in these analyses, we continued to expand our CSIP program to more facilities in our healthcare system and consider it a valuable and necessary part of our infection prevention strategy. Evaluation of CSIP perception of program success is ongoing, switching from evaluation of efficiency and perceived effectiveness to correlating adoption of centralized surveillance with infection-related patient outcomes. Although we are not reporting CSIP job satisfaction, to date CSIP employee retention in our program is very high: through 2022, 5 of 6 CSIPs have remained in the role, with 1 employee transitioning to infection prevention management within the organization. Reduction in HAI is the ultimate objective of this new model of HAI surveillance and LIP work structure.

This investigation had additional limitations. The time spent performing surveillance was self-reported and may have been subject to recall or reporting biases. Surveillance efficiency may have been affected by the complexity of patient cases and distribution of medical conditions, which was not reflected in the summary estimate of efficiency presented here. However, an established measure of HAI surveillance efficiency has not been established. For this analysis, we did not measure the accuracy of HAI surveillance, which may affect the interpretation of time and efficiency findings.

Implementation of a CSIP program leads to more uniform HAI surveillance practices and presents an operational challenge in that selecting strong CSIP candidates and transitioning LIPs to a different role is required. We cannot yet conclude on the effect of this model to improve patient safety. Adoption of centralized surveillance programs have not been widely reported, and there is no accepted approach for doing so. Based on our experience, we provide insights for health systems planning adoption of centralized surveillance (Supplementary Appendix Box). CSIP team members may be empowered to play a role informing and potentially overseeing HAI prevention in multiple facilities. As health system centralized infection prevention-related processes are increasingly implemented, the quality and safety community should maintain a focus on the goal of safe and effective healthcare. In this evaluation, we have begun to explore the hypothetical benefit of centralized surveillance on HAI reduction, but more robust and causal analyses are needed.

The volume and complexity of the infections encountered in centralized surveillance is often not appreciated by the new CSIPs until they receive a full assignment. CSIP staff members are provided with resources and training specific to their roles that emphasizes their previous infection preventionist experience. (For a detailed summary of CSIP onboarding and resources used at our institution, see the Supplementary Appendix.) New CSIP staff members are partnered with another CSIP to be a resource for frequent check-ins and questions and to assist with the volume of work. With a variety of facilities and patient populations across the health system, assignments are balanced in both complexity and volume. The entire department provides peer review of HAI surveillance, documentation, and reporting; monthly calls review areas for improvement, review individual work volume, and highlight successes.

In this quality improvement project report, we demonstrated that a CSIP program for HAI surveillance eases the burden of surveillance on LIPs, allows for increased LIP job satisfaction, and allows for a more stable rate of surveillance and HAI reporting. Future investigations should continue to characterize in these and other novel ways the potential for CSIP programs to improve infection-related patient safety.

## Supplementary material

For supplementary material accompanying this paper visit http://doi.org/10.1017/ash.2023.126. click here to view supplementary material
