# Supplementary material for: Development and implementation of a centralized surveillance infection prevention program in a multi-facility health system: A quality improvement project
Source: Antimicrob Steward Healthc Epidemiol. 2023 Mar 22;3(1):e56. doi: 10.1017/ash.2023.126 (PMC10031579; doi:10.1017/ash.2023.126)
Supplement: Supplementary file 1 [file S2732494X23001262sup.zip › S2732494X23001262sup001.docx]

**Supplemental Table S1: LIP and Unit Director CSIP Response Rates**

**Supplemental Table S1a: Local Infection Preventionist Response Rates**

| **Facility Identifier*** | **Date Pre-CSIP Survey Distributed** | **Response rate (total responses/total sent)** | **Date Post-CSIP Survey Distributed** | **Response rate (total responses/total sent)** |
| --- | --- | --- | --- | --- |
| C1 | 2/20/2019 | 100% (7/7) | 8/21/2019 | 83.3% (5/6) |
| C2 | 4/25/2019 | 100% (5/5) | 12/2/2019 | 100% (5/5) |
| C3 | 8/13/2019 | 100% (1/1) | 4/29/2020 | 100% (1/1) |
| C4 | 8/13/2019 | 100% (1/1) | 4/29/2020 | 100%  (1/1) |
| C5 | 12/5/2019 | 0% (0/1) | N/A | N/A |
| C6 | 12/4/2019 | 100% (1/1) | 10/21/2020 | 0% (0/1) |

**Supplemental Table S1b: Unit Director Response Rates**

| **Facility Identifier*** | **Date Pre-CSIP Survey Distributed** | **Total Count of Surveys Sent** | **Date Post-CSIP Survey Distributed** | **Response rate (total responses/total sent)** |
| --- | --- | --- | --- | --- |
| C1 | 5/6/2019 | 69.4% (25/36) | 4/29/2020 | 62.5% (15/24) |
| C2 | 8/19/2019 | 56.0% (14/25) | 4/29/2020 | 60.0% (9/15) |
| C3 | 8/22/2019 | 87.5% (7/8) | 4/29/2020 | 71.4% (5/7) |
| C4 | 8/22/2019 | 80.0% (4/5) | 4/29/2020 | 25.0% (1/4) |
| C5 | 12/4/2019 | 50% (1/2) | 10/21/2020 | 0.0% (0/1) |
| C6 | 12/4/2019 | 85.7% (6/7) | 10/21/2020 | 33.33% (2/6) |

Abbreviations: LIP – local infection preventionist, CSIP – central surveillance infection prevention

* Due to staffing demands during the COVID-19 pandemic, the surveys were not administered to Facility C7 and Facility C8. Facilities C9 – C12 had not yet implemented CSIP during the survey period and thus were also excluded.

**Supplemental Table S2: Unmatched LIP and UD Pre- and Post-CSIP Implementation Survey Responses**

**Supplemental Table S2a: Unmatched LIP Survey Responses**

| **Survey Question** | **Pre-CSIP Implementation (N=16)** | **Post-CSIP Implementation (N=13)** |
| --- | --- | --- |
| Time allocation* |  |  |
| I spend an adequate amount of time per month on my assigned INPATIENT units | 12.5% | 76.9% |
| I spend an adequate amount of time per month on my assigned ANCILLARY units. | 12.5% | 46.2% |
| I am a knowledgeable resource for providers in my assigned areas. | 75% | 84.6% |
| I am ________ with my involvement in infection control practices in my assigned areas. | 37.5% | 58.3% |
| Infection prevention task satisfaction** |  |  |
| Rate the IP responsibilities according to how rewarding you  find the following tasks | | |
| HAI surveillance competency work | 70 (60.75-80) | 63.5 (36.25-77.75) |
| Unit rounding | 72.5 (58.75-89.25) | 75 (60-82) |
| Root Cause Analyses (RCA) and Case Reviews | 74 (59.75-80) | 57.5 (46.25-76.75) |
| Attending meetings | 65 (53.75-83.5) | 62.5 (50.75-75.75) |
| Phone calls from external customers (e.g., patients, families, DOH, vendors) | 52.5 (36.75-66.25) | 50 (40-70) |
| Phone calls from internal customers (e.g., UDs, nurses, EVS, physicians) | 76 (56.5-80) | 65 (60-75) |
| Individual projects | 86 (75-90.75) | 84 (75.5-90) |
| Collaborative projects with other departments | 84.5 (75-92) | 79 (74.5-94.5) |
| Urgent/emergent situations | 81.5 (69-90) | 71 (38-80) |
| Education (e.g. RNO/PCT orientation, unit-based) | 85 (70.75-90.75) | 70 (51-90) |
| Regulatory tasks (e.g., Joint Commission tracers/audits) | 75 (63-77.5) | 36 (30-70) |
| Quality and professional goal* |  |  |
| As an IP, I have a positive impact on the health system as an organization. | 81.25% | 61.5% |
| My job makes good use of my skills and abilities. | 93.75% | 61.5% |
| I have clearly defined professional goals. | 75% | 46.15% |
| I have enough time to achieve my professional goals. | 43.75% | 46.15% |
| I have clearly defined quality goals. | 68.75% | 69.23% |
| I have enough time to achieve my quality goals. | 37.5% | 53.85% |
| My quality goals are oriented toward improving patient care. | 93.75% | 84.62% |

***** Percent reporting “Agree” or “Strongly Agree”, or “Satisfied” or “Very Satisfied”

** Median (interquartile range) response, with 0 representing “Not Rewarding”, 50 representing “Somewhat Rewarding”, and 100 representing “Very Rewarding”

**Supplemental Table S2b: Unmatched UD Survey Responses**

| **Survey Question*** | **Pre-CSIP Implementation (N=71)** | **Post-CSIP Implementation (N=34)** |
| --- | --- | --- |
| The Infection Preventionist (IP) assigned to my unit spends an adequate amount of time per month on the inpatient  unit. | 46.48% | 67.65% |
| The IP assigned to my unit is a knowledgeable resource for the providers on the inpatient unit. | 91.43% | 88.24% |
| The IP assigned to my unit has played an important role in improving patient care on the inpatient unit. | 61.97% | 76.47% |
| Participating in environmental rounding with my IP is value added. | 73.24% | 88.24% |
| I am _________ with my IP’s involvement in infection control practices on my inpatient unit. | 73.24% | 91.18% |

***** Percent reporting “Agree” or “Strongly Agree”, or “Satisfied” or “Very Satisfied”

**Supplemental Table S3: Complete LIP Survey Results**

**Supplemental Table S3a: Pre-CSIP LIP Survey**

| **Survey Question** | **Respondents (N)** | **Strongly disagree (%)** | **Disagree (%)** | **Neutral (%)** | **Agree (%)** | **Strongly Agree (%)** |
| --- | --- | --- | --- | --- | --- | --- |
| I spend an adequate amount of time per month on my assigned INPATIENT units | 16 | 0 | 8 (50) | 6 (37.5) | 2 (13.5) | 0 (0) |
| I spend an adequate amount of time per month on my assigned ANCILLARY units. | 16 | 2 (13.5) | 12 (75.) | 0 (0) | 1 (6.25) | 1 (6.25) |
| I am a knowledgeable resource for providers in my assigned areas. | 16 | 1 (6.25) | 0 (0) | 3 (18.75) | 11 (68.75) | 1 (6.25) |
| **Survey Question** | **Respondents (N)** | **Very Dissatisfied** | **Dissatisfied** | **Neutral** | **Satisfied** | **Very Satisfied** |
| I am ________ with my involvement in infection control practices in my assigned areas. | 16 | 0 (0) | 2 (13.5) | 8 (50.0) | 6 (37.5) | 0 (0) |
| **For the following 11 questions, rate the IP responsibilities according to how rewarding you find the following tasks by using the sliders below.*** | **Respondents (N)** | **Minimum** | **25%** | **Median** | **75%** | **Maximum** |
| Rate the IP responsibilities according to how rewarding you  find the following tasks |  |  |  |  |  |  |
| HAI surveillance | 14 | 50 | 60.75 | 70 | 80 | 86 |
| Unit rounding | 16 | 15 | 58.75 | 72.5 | 89.25 | 100 |
| Root Cause Analyses (RCA) and Case Reviews | 16 | 49 | 59.75 | 74 | 80 | 100 |
| Attending meetings | 16 | 25 | 53.75 | 65 | 83.5 | 94 |
| Phone calls from external customers (e.g., patients, families, DOH, vendors) | 16 | 5 | 36.75 | 52.5 | 66.25 | 90 |
| Phone calls from internal customers (e.g., UDs, nurses, EVS, physicians) | 16 | 50 | 56.5 | 76 | 80 | 90 |
| Individual projects | 16 | 48 | 75 | 86 | 90.75 | 100 |
| Collaborative projects with other departments | 16 | 60 | 75 | 84.5 | 92 | 100 |
| Urgent/emergent situations | 16 | 31 | 69 | 81.5 | 90 | 100 |
| Education (e.g. RNO/PCT orientation, unit-based) | 16 | 30 | 70.75 | 85 | 90.75 | 100 |
| Regulatory tasks (e.g., Joint Commission tracers/audits) | 3 | 51 | 63 | 75 | 77.5 | 80 |
| **Survey Question** | **Respondents (N)** | **Strongly disagree (%)** | **Disagree (%)** | **Neutral (%)** | **Agree (%)** | **Strongly Agree (%)** |
| As an IP, I have a positive impact on the health system as an organization. | 16 | 0 (0) | 0 (0) | 2 (12.5) | 11 (68.75) | 3 (18.75) |
| My job makes good use of my skills and abilities. | 16 | 0 (0) | 0 (0) | 1 (6.25) | 12 (75.0) | 3 (18.75) |
| I have clearly defined professional goals. | 16 | 0 (0) | 1 (6.25) | 3 (18.75) | 9 (56.25) | 3 (18.75) |
| I have enough time to achieve my professional goals. | 16 | 1 (6.25) | 2 (12.5) | 6 (37.5) | 7 (43.75) | 0 (0) |
| I have clearly defined quality goals. | 16 | 0 (0) | 1 (6.25) | 4 (25.0) | 8 (50.0) | 3 (18.75) |
| I have enough time to achieve my quality goals. | 16 | 0 (0) | 5 (31.25) | 5 (31.25) | 6 (37.5) | 0 (0) |
| My quality goals are oriented toward improving patient care. | 16 | 0 (0) | 0 (0) | 1 (6.25) | 9 (56.25) | 6 (37.5) |

*******Median response, with 0 representing “Not Rewarding”, 50 representing “Somewhat Rewarding”, and 100 representing “Very Rewarding”

**Supplemental Table S3b: Post-CSIP LIP Survey**

| **Please select tasks on which you now spend more time and effort in place of surveillance. Check all that apply.** | **Percent (number) responding (N=X)** |
| --- | --- |
| Attending meetings | 69.2% (N=9) |
| Additional individual projects | 61.5% (N=8) |
| Additional collaborative projects | 69.2% (N=9) |
| Studying or reading infection prevention-related topics | 23.1% (N=3) |
| Increasing time on assigned INPATIENT units | 84.6% (N=11) |
| Increasing time on assigned ANCILLARY units | 46.2% (N=6) |
| Strengthening professional relationships outside of the IP department | 53.8% (N=7) |
| Attending more hospital events (i.e. fairs, talks, luncheons, etc.) | 30.8% (N=4) |

| **Survey Question** | **Respondents (N)** | **Strongly disagree (%)** | **Disagree (%)** | **Neutral (%)** | **Agree (%)** | **Strongly Agree (%)** |
| --- | --- | --- | --- | --- | --- | --- |
| I spend an adequate amount of time per month on my assigned INPATIENT units | 13 | 0 (0) | 0 (0) | 3 (23.08) | 9 (69.23) | 1 (7.69) |
| I spend an adequate amount of time per month on my assigned ANCILLARY units. | 13 | 0 (0) | 4 (30.77) | 3 (23.08) | 6 (46.15) | 0 (0) |
| I am a knowledgeable resource for providers in my assigned areas. | 13 | 0 (0) | 0 (0) | 2 (15.38) | 8 (61.54) | 3 (23.08) |
| **Survey Question** | **Respondents (N)** | **Very Dissatisfied (%)** | **Dissatisfied (%)** | **Neutral (%)** | **Satisfied (%)** | **Very Satisfied (%)** |
| I am ________ with my involvement in infection control practices in my assigned areas. | 12 | 0 | 0 | 5 | 5 | 2 |
| **For the following 11 questions, rate the IP responsibilities according to how rewarding you find the following tasks by using the sliders below.*** | **Respondents (N)** | **Minimum** | **25%** | **Median** | **75%** | **Maximum** |
| Rate the IP responsibilities according to how rewarding you  find the following tasks |  |  |  |  |  |  |
| HAI surveillance | 10 | 18 | 36.25 | 63.50 | 77.75 | 85 |
| Unit rounding | 13 | 21 | 60 | 75 | 82 | 100 |
| Root Cause Analyses (RCA) and Case Reviews | 12 | 17 | 46.25 | 57.5 | 76.75 | 86 |
| Attending meetings | 12 | 20 | 50.75 | 62.5 | 75.75 | 86 |
| Phone calls from external customers (e.g., patients, families, DOH, vendors) | 13 | 0 | 40 | 50 | 70 | 86 |
| Phone calls from internal customers (e.g., UDs, nurses, EVS, physicians) | 13 | 26 | 60 | 65 | 75 | 95 |
| Individual projects | 10 | 19 | 75.50 | 84 | 90 | 95 |
| Collaborative projects with other departments | 12 | 38 | 74.5 | 79 | 94.5 | 100 |
| Urgent/emergent situations | 13 | 32 | 38 | 71 | 80 | 90 |
| Education (e.g. RNO/PCT orientation, unit-based) | 13 | 27 | 51 | 70 | 90 | 100 |
| Regulatory tasks (e.g., Joint Commission tracers/audits) | 13 | 0 | 30 | 36 | 70 | 90 |
| **Survey Question** | **Respondents (N)** | **Strongly disagree (%)** | **Disagree (%)** | **Neutral (%)** | **Agree (%)** | **Strongly Agree (%)** |
| As an IP, I have a positive impact on the health system as an organization. | 13 | 0 (0) | 0 (0) | 5 (38.46) | 6 (46.15) | 2 (15.38) |
| My job makes good use of my skills and abilities. | 13 | 2 (15.38) | 0 (0) | 3 (23.08) | 4 (30.77) | 4 (30.77) |
| I have clearly defined professional goals. | 13 | 1 (7.69) | 1 (7.69) | 5 (38.46) | 4 (30.77) | 2 (15.38) |
| I have enough time to achieve my professional goals. | 12 | 0 (0) | 1 (8.33) | 5 (41.67) | 4 (33.33) | 2 (16.67) |
| I have clearly defined quality goals. | 13 | 0 (0) | 0 (0) | 4 (30.77) | 9 (69.23) | 0 (0) |
| I have enough time to achieve my quality goals. | 13 | 0 (0) | 0 (0) | 6 (46.15) | 7 (53.85) | 0 (0) |
| My quality goals are oriented toward improving patient care. | 13 | 0 (0) | 0 (0) | 2 (15.38) | 6 (46.15) | 5 (38.46( |

*******Median response, with 0 representing “Not Rewarding”, 50 representing “Somewhat Rewarding”, and 100 representing “Very Rewarding”

**Supplemental Figure S1: Reallocation of LIP time**


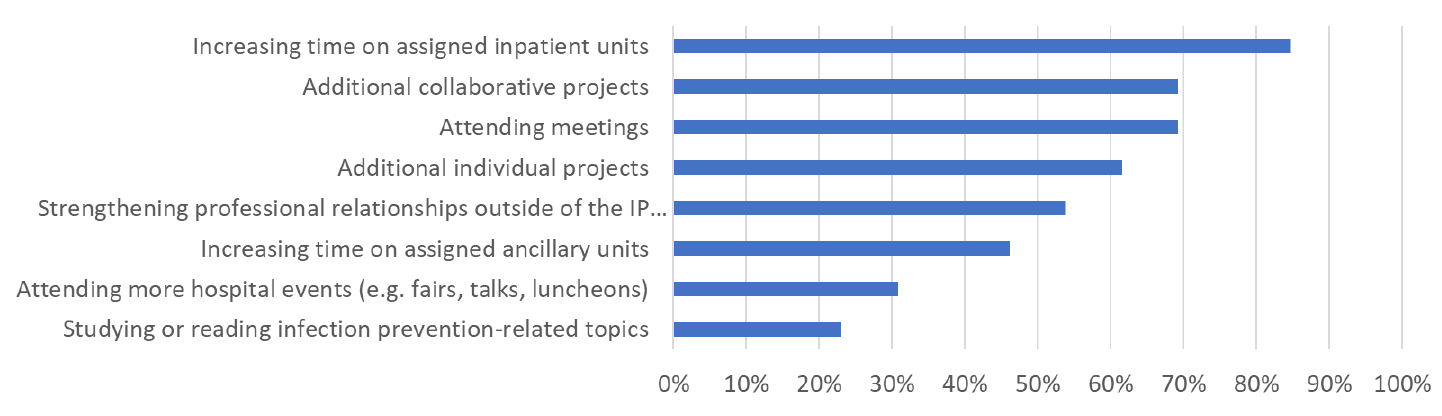


Figure S1: LIPs were asked which of their normal duties they were able to devote more time to after the implementation of CSIP. Answers are shown as a percentage of LIPs (N=13) who indicated they were able to spend more time on the indicated task. Abbreviations: LIP – local infection preventionist

**Supplemental Table S4: Complete UD Survey Results**

**Supplemental Table S4a: Pre-CSIP UD Survey**

| **Survey Question** | **Respondents (N)** | **Strongly disagree (%)** | **Disagree (%)** | **Neutral (%)** | **Agree (%)** | **Strongly Agree (%)** |
| --- | --- | --- | --- | --- | --- | --- |
| The Infection Preventionist (IP) assigned to my unit spends an adequate amount of time per month on the inpatient  unit. | 71 | 2 (2.82) | 13 (18.31) | 23 (32.39) | 22 (31.00) | 11 (15.49) |
| The IP assigned to my unit is a knowledgeable resource for the providers on the inpatient unit. | 70 | 1 (1.43) | 2 (2.86) | 3 (4.29) | 30 (42.86) | 34 (48.57) |
| The IP assigned to my unit has played an important role in improving patient care on the inpatient unit. | 71 | 1 (1.41) | 9 (12.68) | 17 (23.94) | 29 (40.84) | 15 (21.13) |
| Participating in environmental rounding with my IP is value added. | 71 | 2 (2.82) | 4 (5.63) | 13 (18.31) | 31 (43.66) | 21 (29.58) |
| **Survey Question** | **Respondents (N)** | **Very Dissatisfied (%)** | **Dissatisfied (%)** | **Neutral (%)** | **Satisfied (%)** | **Very Satisfied (%)** |
| I am _________ with my IP's involvement in infection control practices on my inpatient unit. | 71 | 1 (1.41) | 2 2 (2.82) | 16 (22.54) | 27 (38.03) | 25 (35.21) |

| **Open-Ended Answers** | **Responses: Two most prevalent themes with examples (theme frequency among all responses)** |
| --- | --- |
| What are 2 ways in which the IP contributes to improving patient care and safety on your inpatient unit? | Theme: Providing education to unit staff  Theme: IP as a Real-time resource (43.14%)  “Excellent resource for any question myself or staff have. Very quick to respond.”  “…She is also great at being responsive to real-time patient questions.”  “She keeps me updated frequently and deals with issues in real time. Example will be when a wrong isolation sign is hanging, she will educate the staff on the correct type of isolation and the proper isolation sign for it.”  Theme: Environmental Rounding (33.33%)  “I feel the bimonthly rounds keep everyone on their toes and vigilant of the IP's presence. I think we look for affirmation of our practice through rounding and appreciate all feedback when our associates are not following proper protocol. Spot check on signage also helps!”  “Rounds are very important and makes a difference when EVS is with us.” |
| What are 2 ways in which the IP could be more effective at improving patient care and safety on your inpatient unit? | Theme: Increased presence on inpatient unit (52.17%)  “Having more unit presence so that staff are aware of our IP and their role”  “More rounding with staff members, more availability to complete inservices/education”  Theme: More education to staff (47.83%)  “Educational opportunities; assist with intermittent audits to ensure staff compliance with practice”  “1.) Education for the staff during huddles 2.) In-servicing on the unit in partnership with unit leadership” |
| In a few sentences or less, what are your  expectations for your IP? | Theme: Staff education (54.05%)  “Available to teach staff when something new or questions arise”  “Do real time education and coaching on issues that she sees instead of writing an email later. it's helpful for the staff to hear feedback from someone of leadership other than the UD”  Theme: IP as a real-time resource (37.84%)  “For staff to be able to identify her as a real time resource when issues arise. Need to have a IP contact after hours. Difficult to find out who is on call or covering for our unit.”  “To be easily accessible to my team to clarify things when they need clarification” |

**Supplemental Table S4b: Post-CSIP UD Survey**

| **Survey Question** | **Respondents (N)** | **Strongly disagree (%)** | **Disagree (%)** | **Neutral (%)** | **Agree (%)** | **Strongly Agree (%)** |
| --- | --- | --- | --- | --- | --- | --- |
| The Infection Preventionist (IP) assigned to my unit spends an adequate amount of time per month on the inpatient  unit. | 34 | 3 (8.82) | 4 (11.76) | 4 (11.76) | 9 (26.47) | 14 (41.18) |
| The IP assigned to my unit is a knowledgeable resource for the providers on the inpatient unit. | 34 | 3 (8.82) | 0 (0) | 1 (2.94) | 8 (23.53) | 22 (64.71) |
| The IP assigned to my unit has played an important role in improving patient care on the inpatient unit. | 34 | 3 (8.82) | 2 (5.88) | 3 (8.82) | 8 (23.53) | 18 (52.94) |
| Participating in environmental rounding with my IP is value added. | 34 | 4 (11.76) | 0 (0) | 0 (0) | 14 (41.17) | 16 (47.06) |
| **Survey Question** | **Respondents (N)** | **Very Dissatisfied (%)** | **Dissatisfied (%)** | **Neutral (%)** | **Satisfied (%)** | **Very Satisfied (%)** |
| I am _________ with my IP's involvement in infection control practices on my inpatient unit. | 34 | 1 (2.94) | 2 (5.88) | 0 (0) | 10 (29.41) | 21 (61.76) |

| **Open-Ended Answers** | **Responses (theme and verbatim)** |
| --- | --- |
| What are 2 new ways in which the IP contributes to improving patient care and safety on your inpatient unit? | Theme: Education to Staff (64.00%)  “[My IP] provides updates and checks in when we have patients with rare isolation with recommendations as well as sending educational tools/flyers for staff”  “When a consult is needed we have the IP's complete attention for education and direction.”  Theme: Reporting of data (44.00%)  “Our Infection Preventionist regularly presents to our ICU leadership regarding the specific metrics for ICU HAI for every unit at the hospital. She also shares case study examples at our Surg/Onc Quality meetings to help educate on what can be done better both clinically and in documentation.”  “sending monthly[infection] reports” |
| What are 2 ways in which the IP could be more effective at improving patient care and safety on your inpatient unit? | Theme: Nothing – satisfied with IP work (47.37%)  “Nothing but positive experiences with [My IP], she is always accessible and provides resources to staff as needed, unable to identify any suggestions at this time to improve patient care”  “None right now- it's been great.”  Theme: More huddles/education/rounding (21.05%)  “More information or where to get more information on isolation precautions”  “Short Power point educational IP topics for staff . Short huddles with staff. [My IP] has offered and continues to do so.“ |
| In a few sentences, how have your expectations for IP changed since Centralized Surveillance has been in place? | Theme: Increased IP Involvement (60.00%)  “I have definitely felt more involvement in the unit from my IP which has been great.”  “I feel the personalized care and dedication really shows to my staff now more than ever. [My IP] does a really nice job keeping everyone informed and up to date with factual information.”  Theme: More IP accessibility (35.00%)  “Accessibility has improved”  ”Our expectation is that [Our IP] would be more accessible, which has definitely been the case.” |

**Supplemental Figures S2-S4**

For these data, Infection Prevention & Control managers at all 24 facilities were surveyed in May 2021 using the methodology described in the text. The survey was voluntary and confidential, and asked three questions about the impact of the pandemic on Infection Prevention & Control team activities. Twenty-four of managers were invited, and 24 (100%) responded.

**Supplemental Figure S2: Ranking which LIP activities were most negatively impacted by the COVID-19 pandemic**


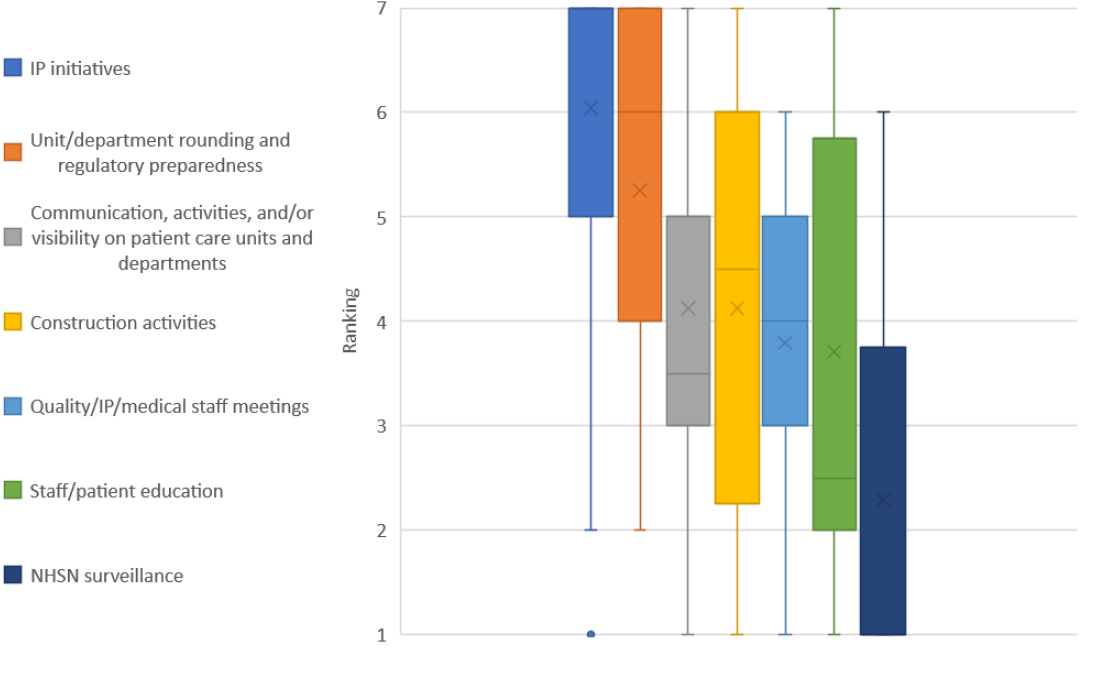


Abbreviations: LIP – local infection preventionist, IP – infection preventionist, NHSN – National Healthcare Safety Network

**Supplemental Figure S3: LIP ranking of job duties that consumed most of their time during the COVID-19 pandemic**


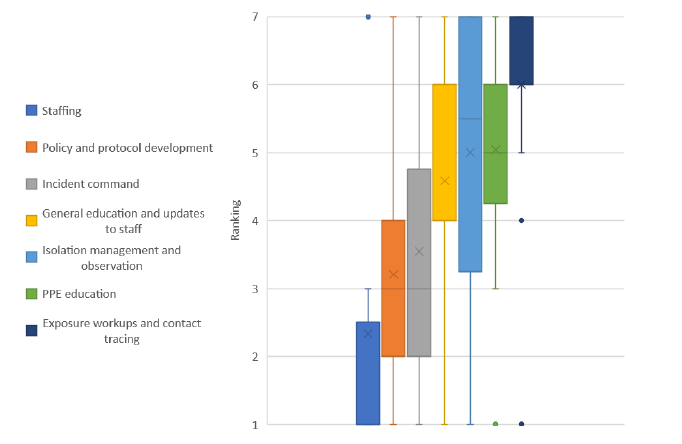


Abbreviations: LIP – local infection preventionist, IP – infection preventionist, PPE – personal protective equipment

**Supplemental Figure S4: LIP initiatives that were delayed or canceled due to the COVID-19 pandemic**


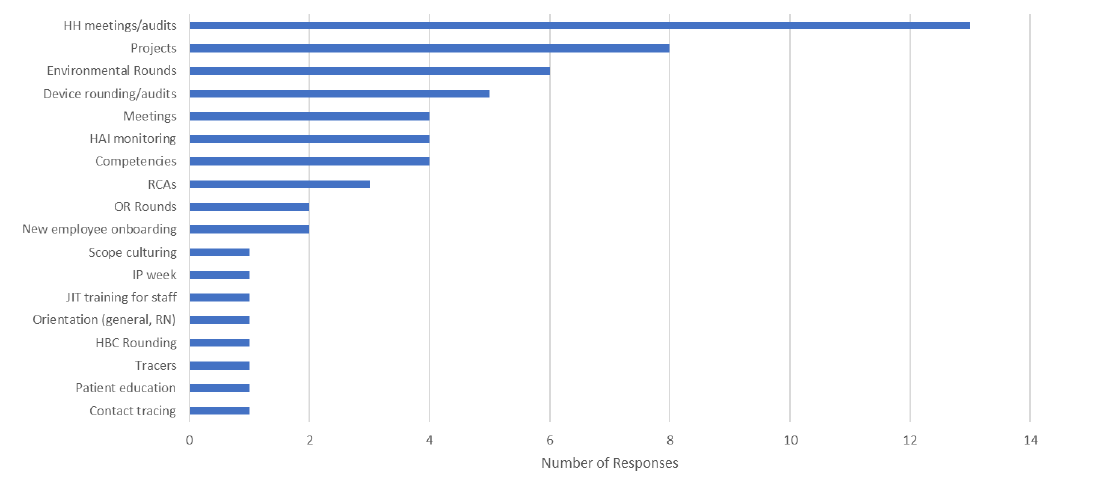


Abbreviations: LIP – local infection preventionist, HH – hand hygiene, HAI – healthcare-associated infection, RCA – root cause analysis, OR – operating room, IP – infection preventionist, JIT – just-in-time, HBC – hospital-based clinic
