# Supplementary material for: Development and implementation of a centralized surveillance infection prevention program in a multi-facility health system: A quality improvement project
Source: Antimicrob Steward Healthc Epidemiol. 2023 Mar 22;3(1):e56. doi: 10.1017/ash.2023.126 (PMC10031579; doi:10.1017/ash.2023.126)

**Supplemental Appendix: CSIP Training and Documentation Overview**

This appendix includes descriptions and examples of training, onboarding, and reference materials given to our system CSIPs. It should be noted that most CSIP policies and documentation procedures (including NHSN, TheraDoc, and RiskMaster documentation) are specific to our health system, and training materials should reflect specific institutional policies. Additionally, specific NHSN documentation training is not provided during CSIP onboarding. CSIP staff members are hired with the expectation of prior infection prevention experience, and thus all training and onboarding materials presume knowledge of NHSN reporting procedures.

CSIP and LIP team members communicate regularly via email, chat messaging, phone calls, and regularly scheduled standing meetings. Regular communication between infection prevention team members is critical for the transition of work and to ensure confidence in CSIP work and reporting. LIPs communicate with frontline workers through regular HAI reports and reports of specific HAI events. We maintain standardization in communication between facilities, as CSIPs cover multiple facilities and maintaining efficiency is our main goal.

**The following are our recommendations for organizations wishing to implement centralized surveillance:**

| **Box. Considerations for health systems adopting centralized surveillance**   - **Recruit staff well-suited to centralized surveillance.** Experienced senior infection preventionists, with a commitment to surveillance fidelity, and who desired a work lifestyle that permitted flexible work-from-home hours, have proven successful in our experience. Challenges faced, especially with a small team, included coverage for CSIP absences, maintaining a level workload, and sustaining timely reporting. CSIPs also need to feel confident yet receptive to questions about HAI adjudication from physicians and other infection preventionists. - **Consider evaluating CSIP surveillance efficiency as well as fidelity to surveillance definitions.** CSIP teams will handle high volumes of infection cases, providing an opportunity to increase efficiency and identify non-valued work. Evaluation of surveillance accuracy may be through CSIP team inter-rater reliability, validation with non-CSIP experience LIPs in the organization, or external auditors such as state public health regulators. - **Incorporating LIP perspective on processes for CSIP communication** of HAI results and notable patterns will assuage LIP concerns they may be missing significant events or losing familiarity with the data. An open line of communication between CSIP team members maintains collaborative evaluation of complex cases. - **Create standardized reports for HAI events**, including templated data visualizations for internal HAI reporting (e.g., leadership). - **Anticipate changes in job description and desirability for LIPs.** Converting time spent doing surveillance to outcomes-oriented project-based and more inter-disciplinary work will require preparation, support, and potentially training for some LIPs. LIPs who find computer-based technical surveillance gratifying may be dissatisfied with a greater emphasis on quality improvement. LIPs Many LIPs will also seek continued education and experience with HAI surveillance to maintain skills for positions outside of the organization. - **Anticipate a “business case” for adoption of centralized surveillance**. It may be difficult to convince administrators to fund additional full-time employee positions for a function completed by existing staff. Health system infection prevention and control leaders have a role to play with local infection prevention leaders in demonstrating that LIP time is effectively re-allocated to more effective HAI reduction with demonstrable outcomes in patient safety. |
| --- |

**The following documents are provided to new CSIP staff members:**

- ICD-10 and procedure codes for all operative procedures for NHSN documentation, including guidance to for specific NHSN documentation of hip prosthesis and knee prosthesis operative procedures
- A list of all active facilities and their common abbreviations within our health system
- Comprehensive contact information for appropriate individuals at various facilities, including, but not limited to, local infection preventionists, medical directors, administrative liaisons, lab service directors, point of care personnel, quality/regulatory staff, automated testing lab staff, histology lab, histocompatibility lab, immunopathology lab, hematology lab, microbiology lab, and virology lab staff.
- Tip sheets outlining the procedure for HAI review processes at the facilities where CSIPs will be responsible for HAI surveillance
- Detailed instructions for reporting HAI via TheraDoc software


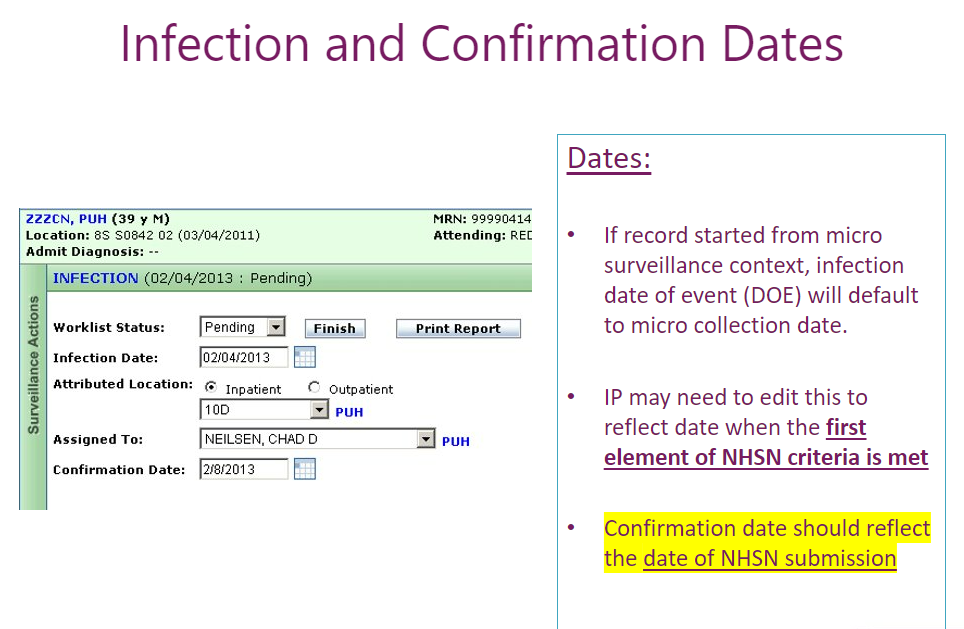


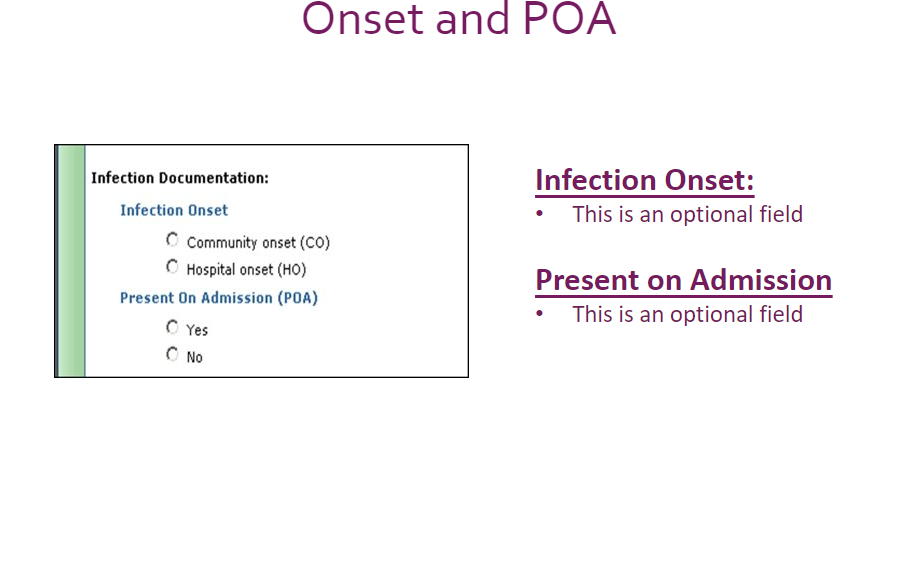


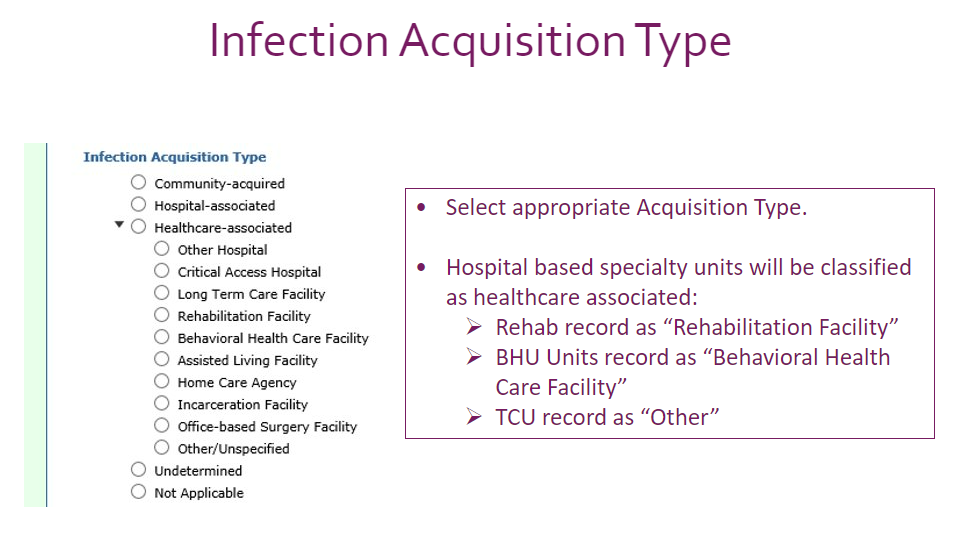


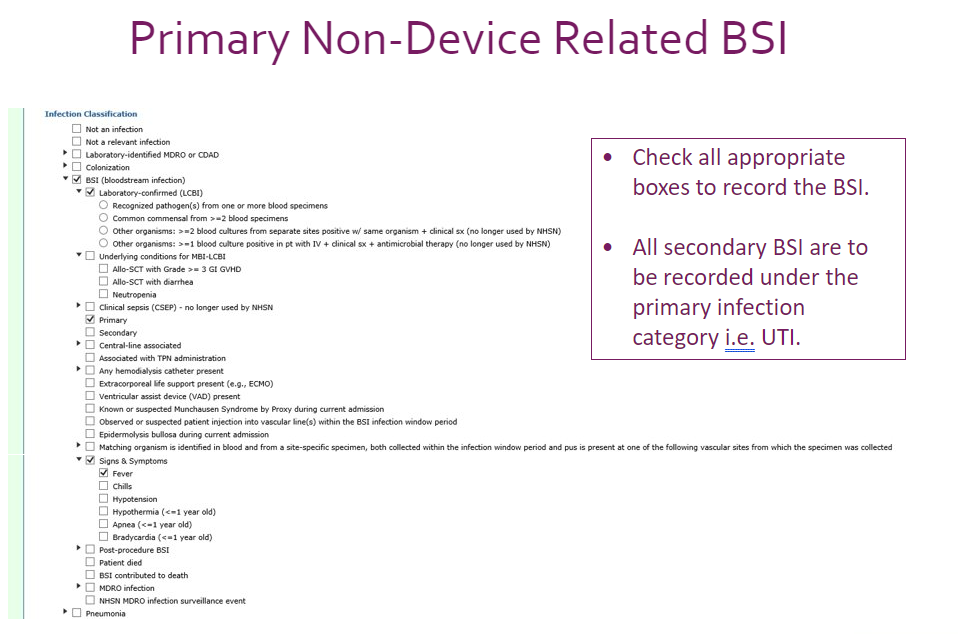


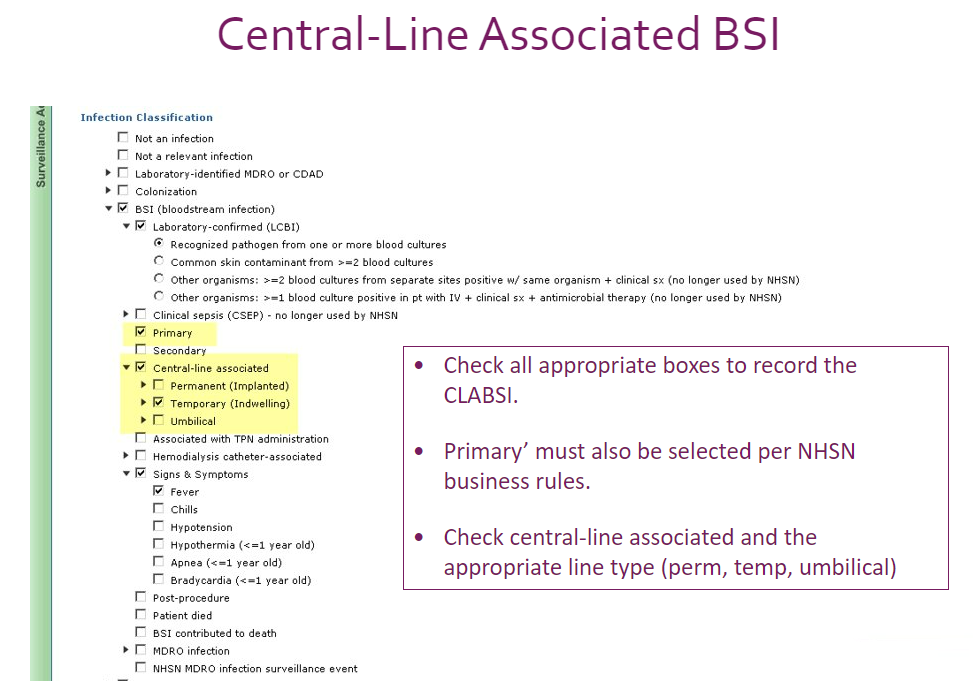


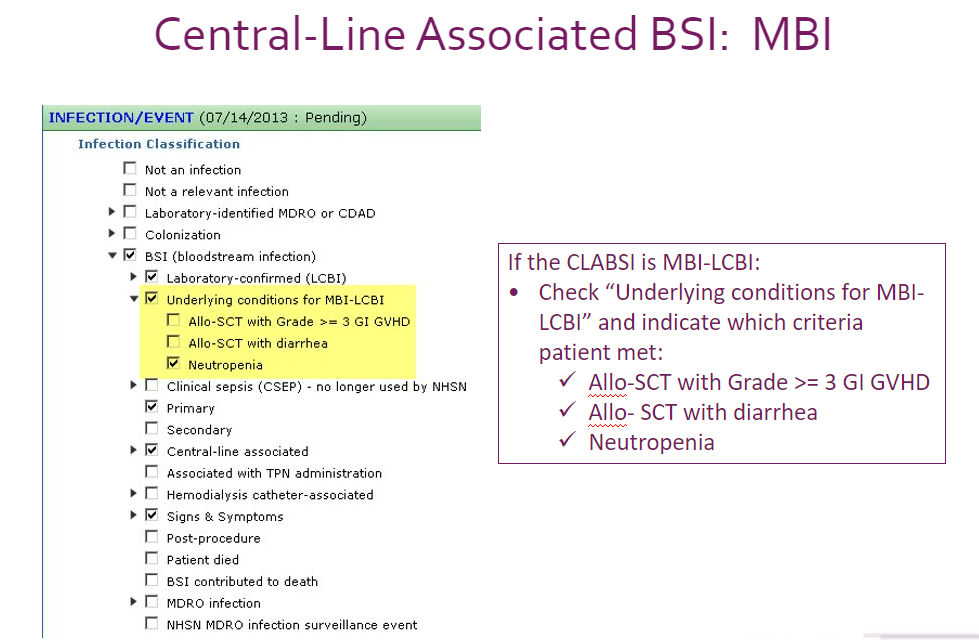


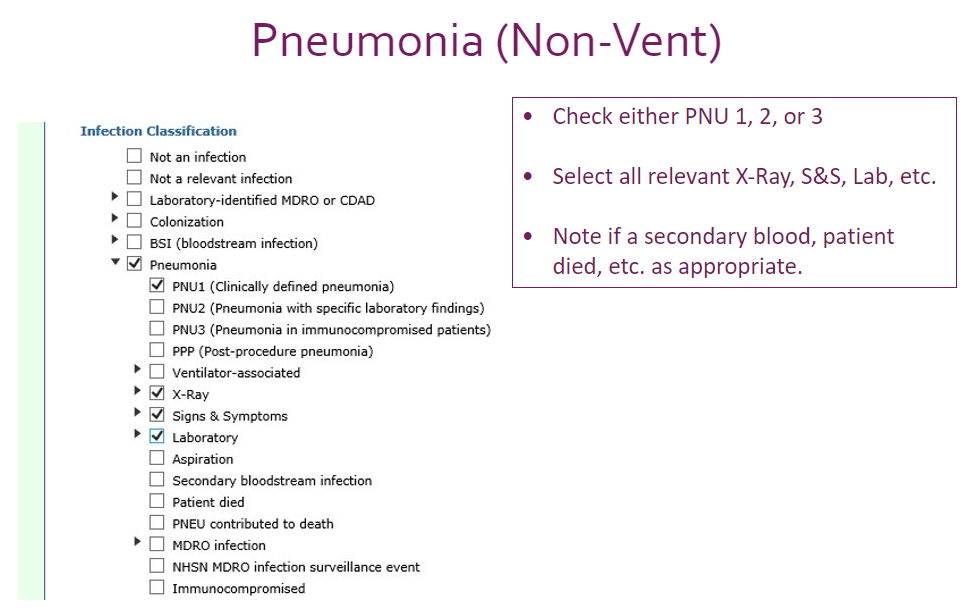


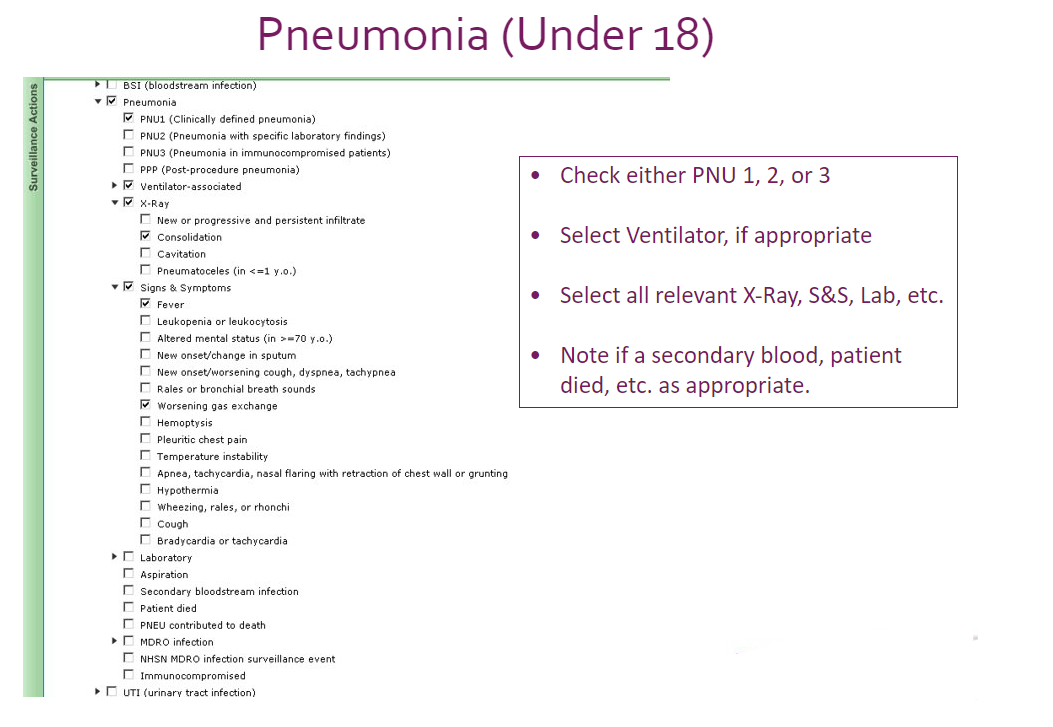


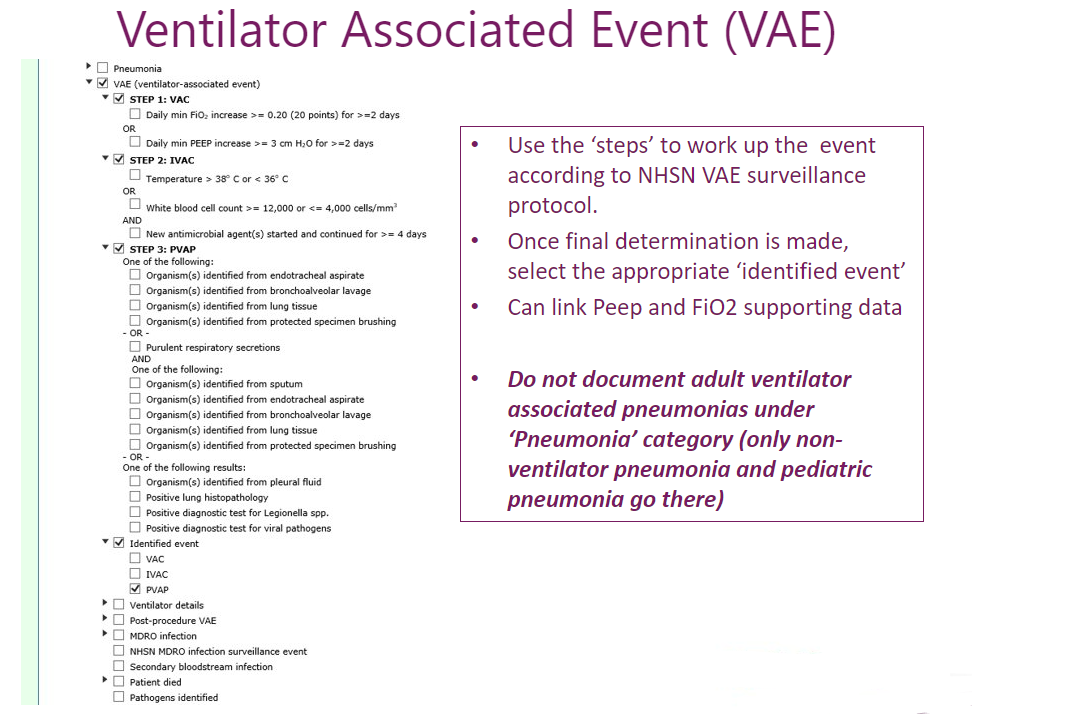


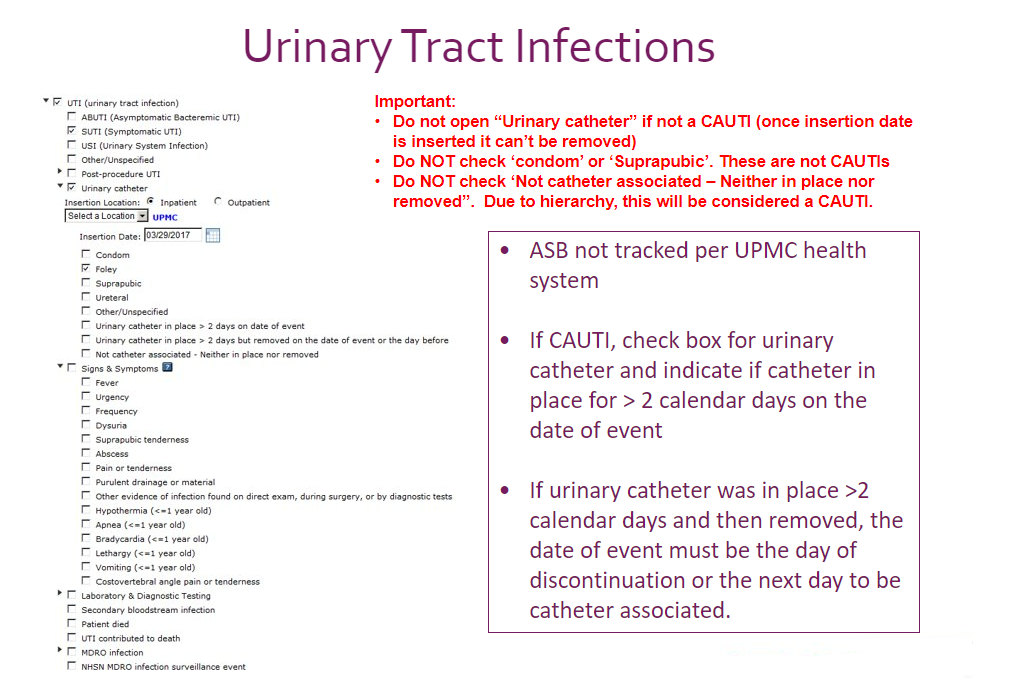


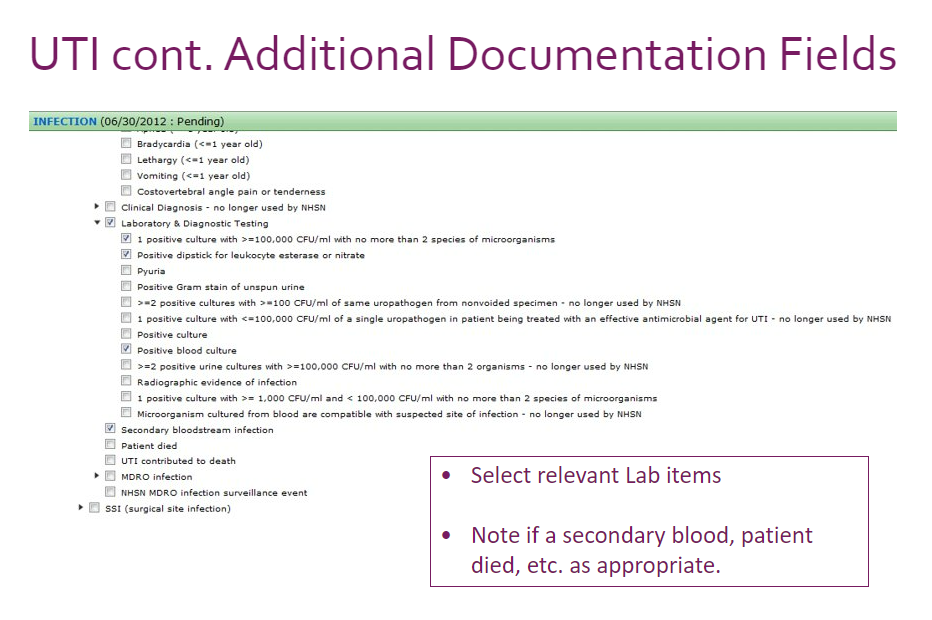


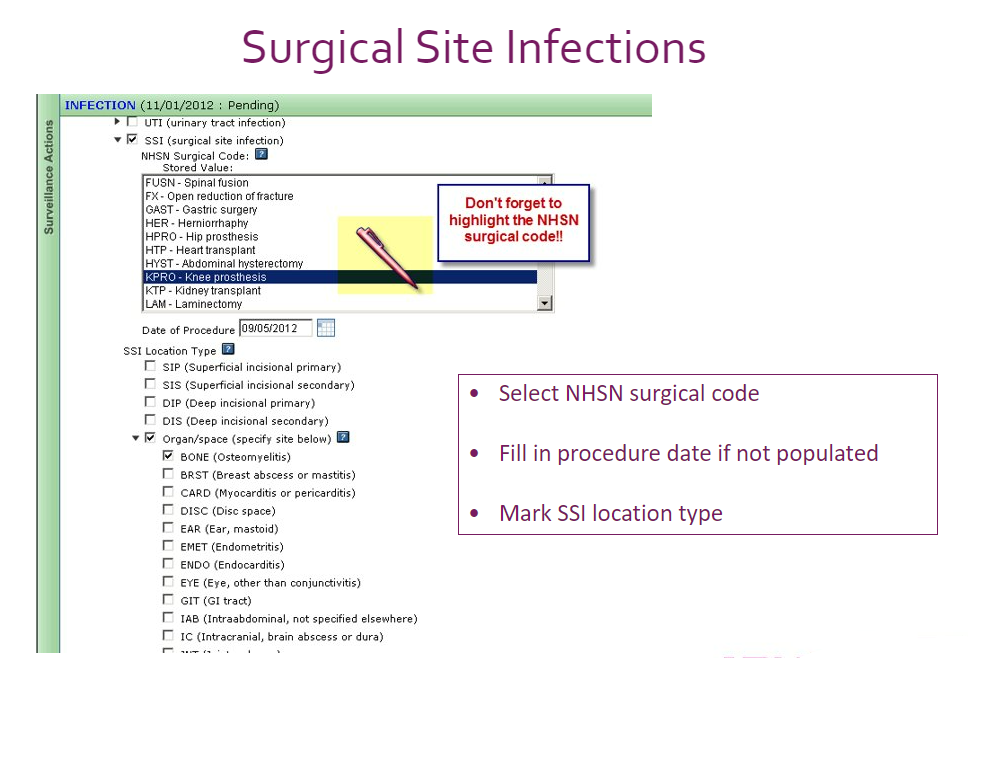


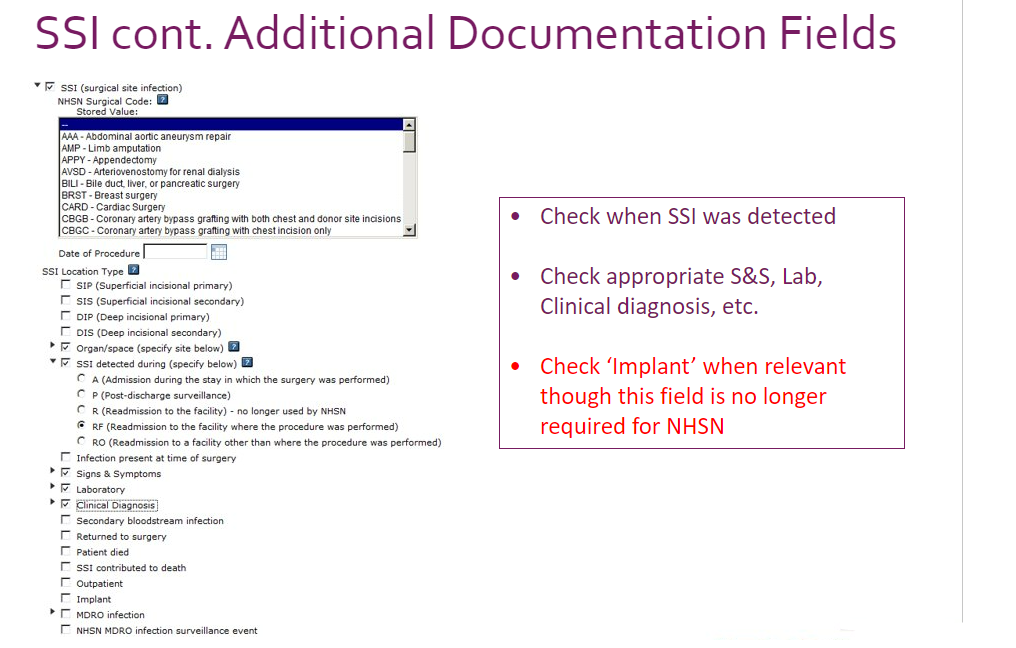


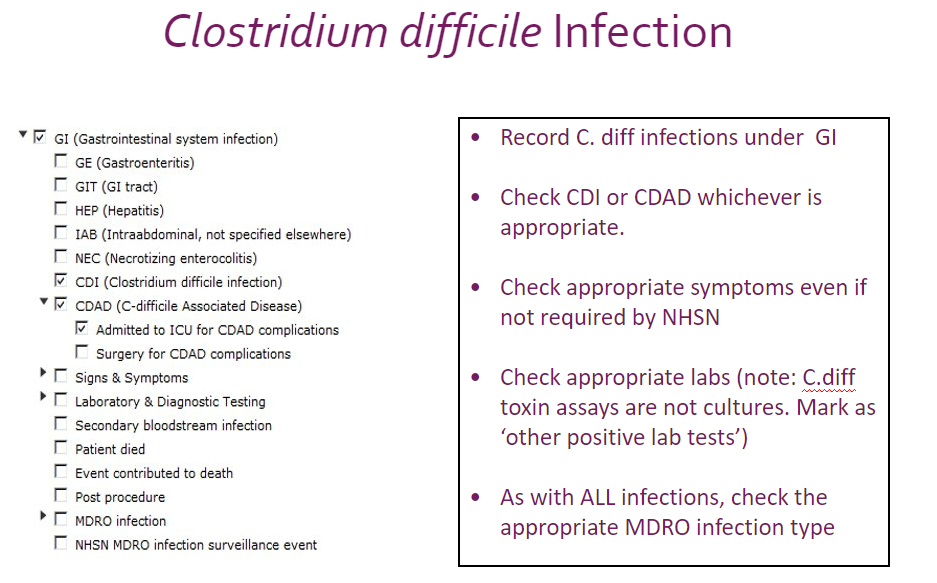


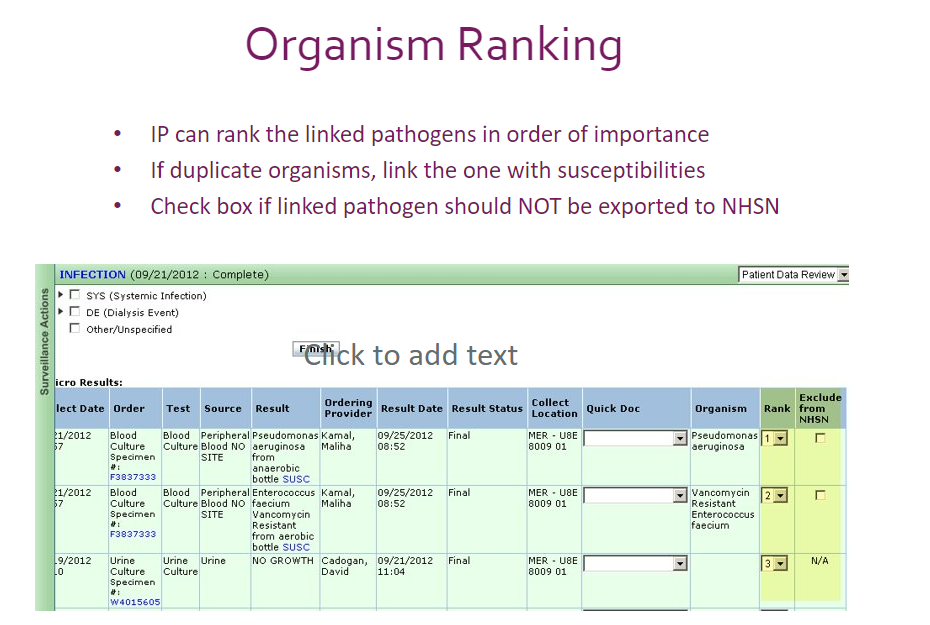


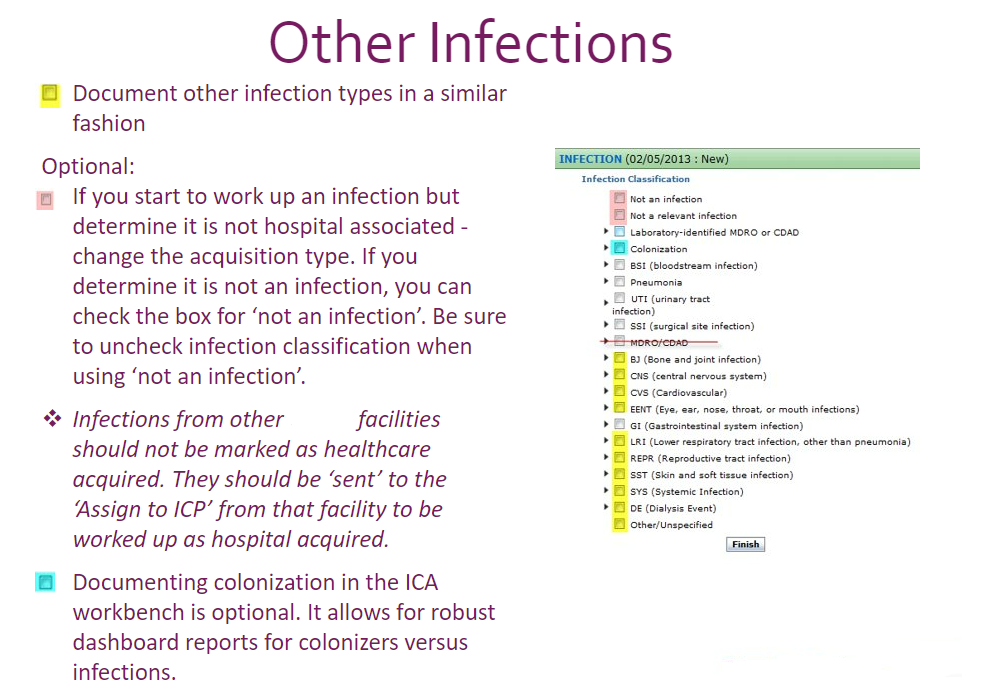


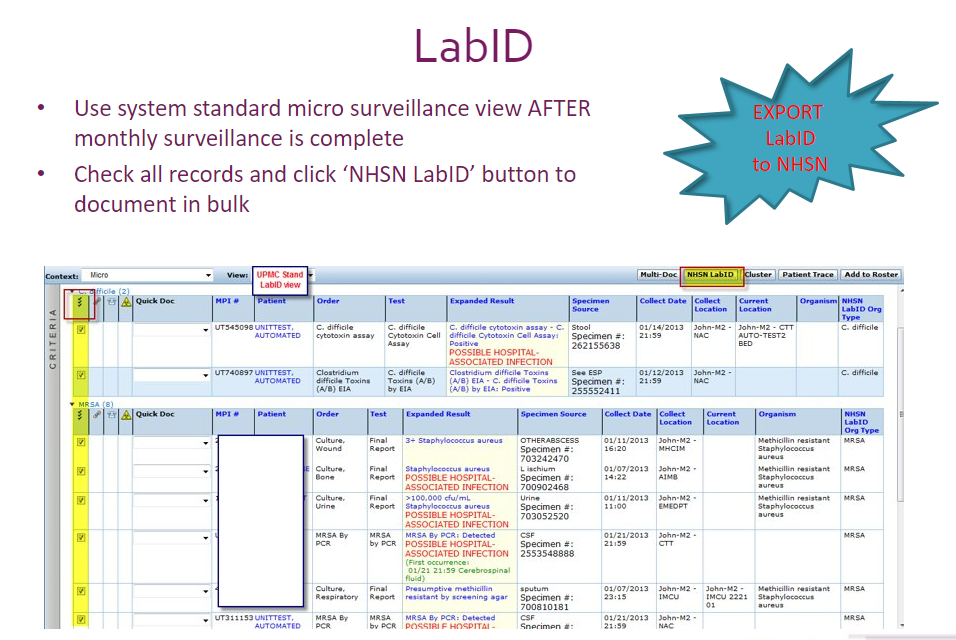


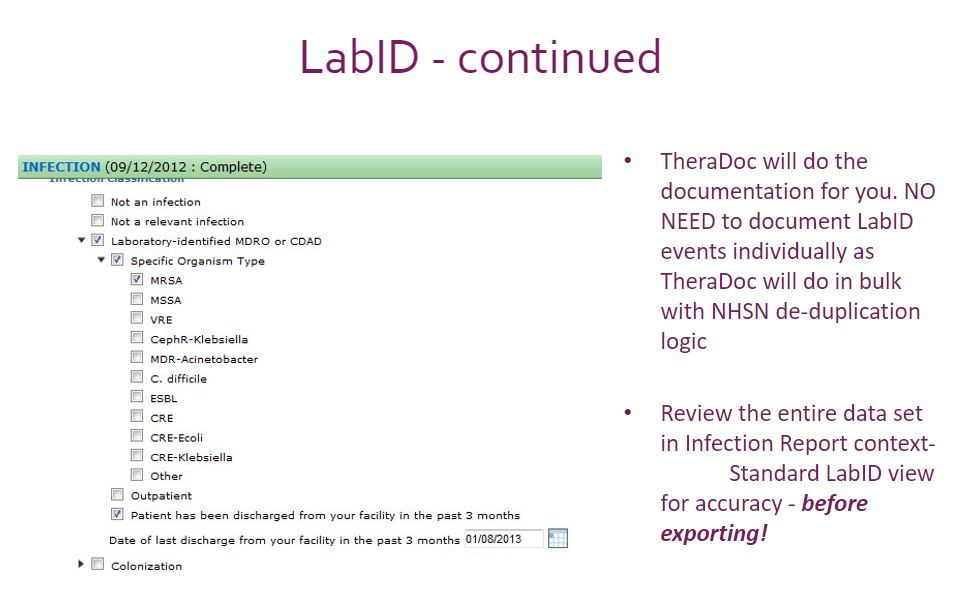


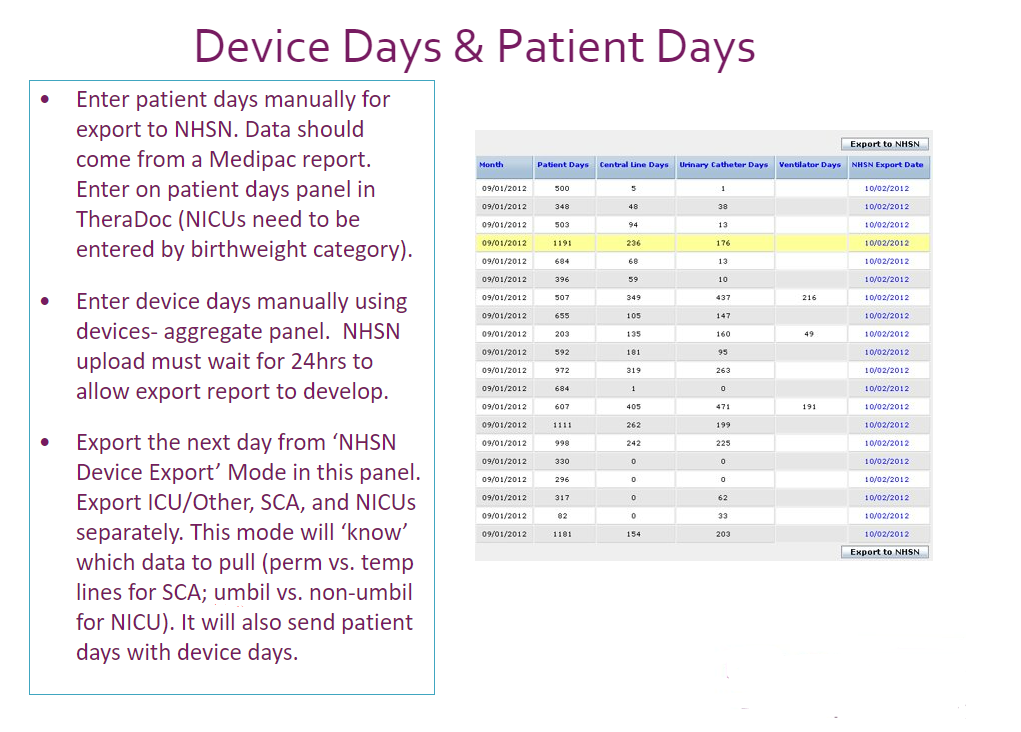


**The following resources are given to CSIPs to use in their daily work:**

- CSIP TheraDoc Quick Doc Reference Guide: documentation how-to for new CSIPs, specific to TheraDoc clinical decision support software


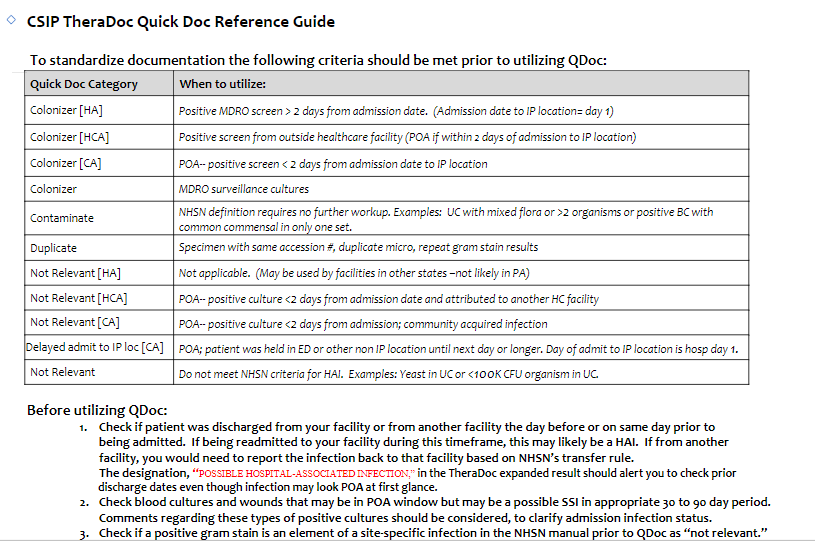


- CSIP Response to Physician Inquiry regarding HAI determination: template for CSIPs to respond to physician inquiries about a specific HAI case
- CSIP RiskMaster documentation – info to include when documenting a HAI in the incident reporting software RiskMaster


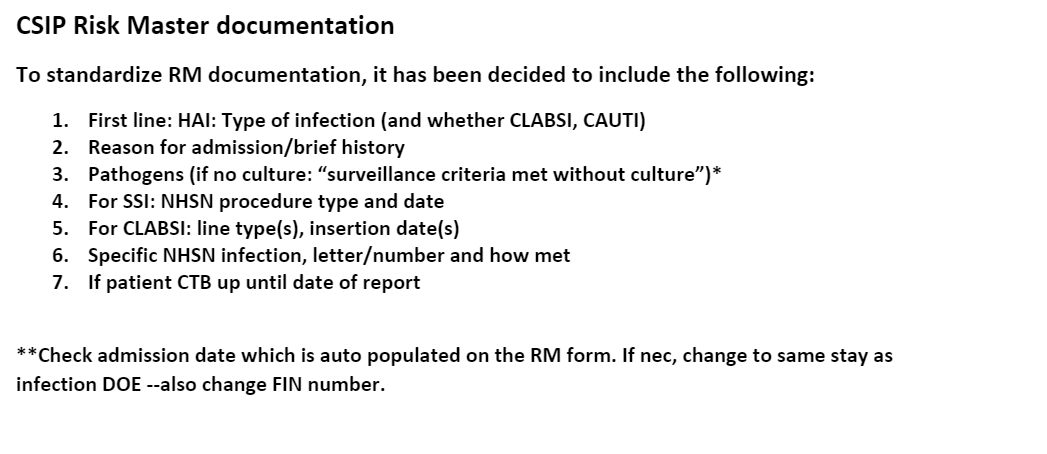


- Information for documenting infections present at the time of surgery


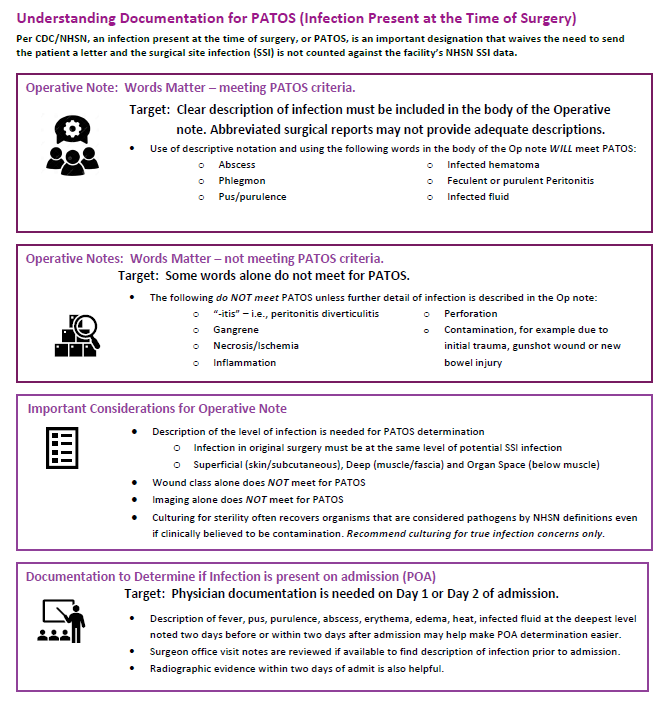


**This presentation is given when a facility is transitioning to CSIP:**


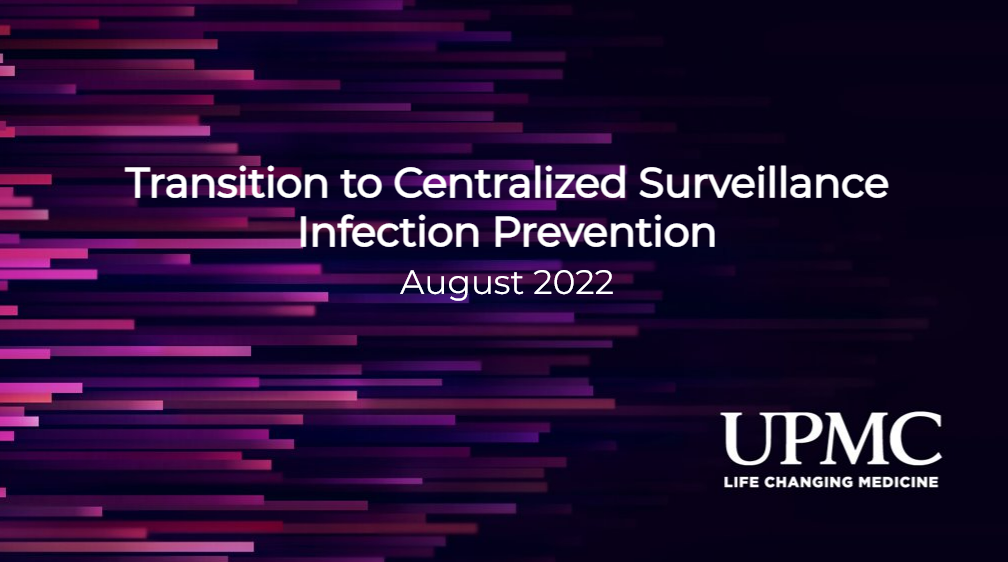


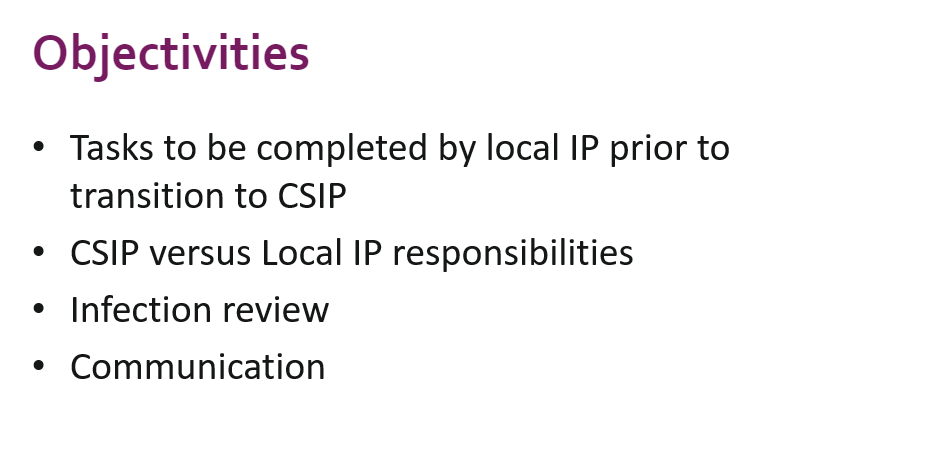


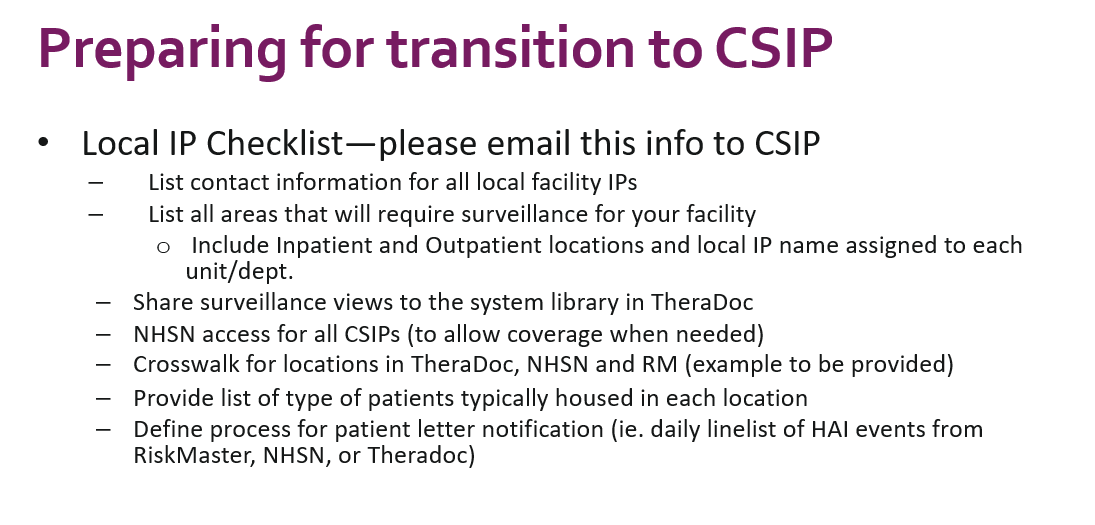


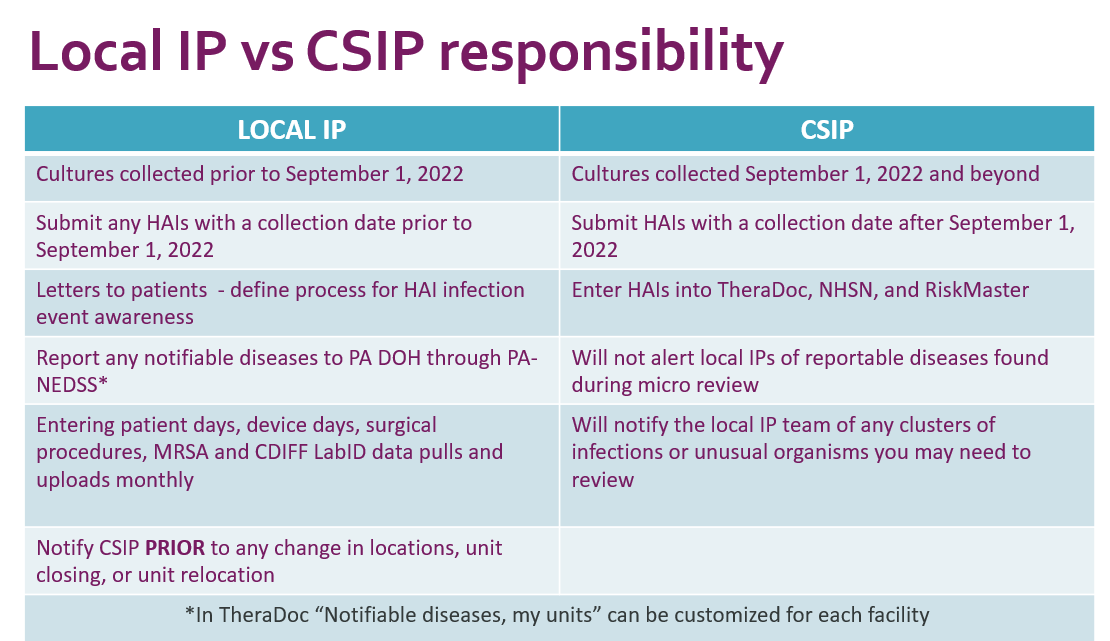


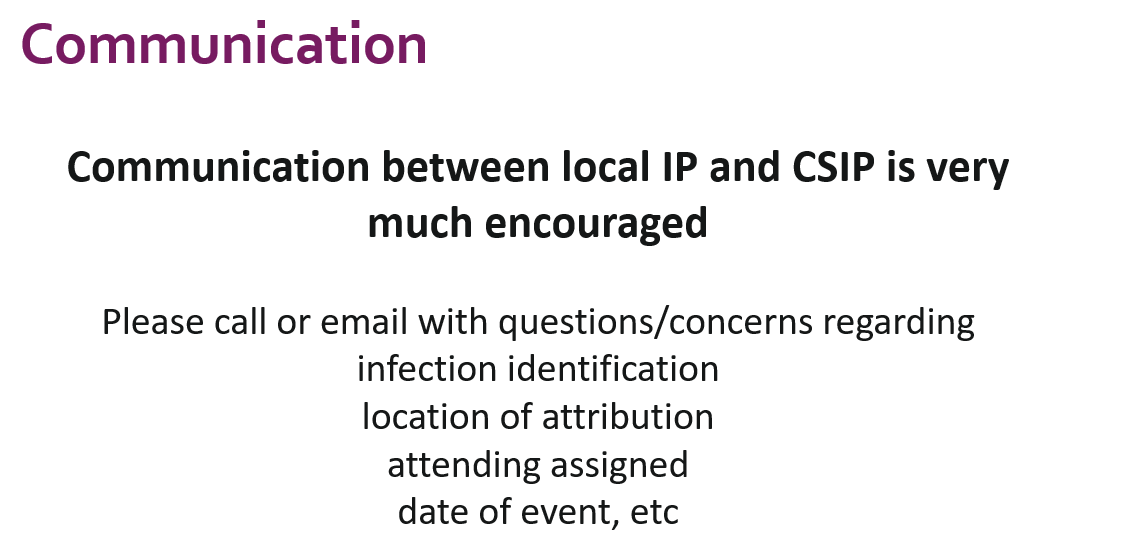

Supplement: Supplementary file 1 [file S2732494X23001262sup.zip › S2732494X23001262sup002.docx]
